# Cationic Peptoids for Systemic In Vivo Cartilage‐Targeting

**DOI:** 10.1002/advs.202502781

**Published:** 2025-08-31

**Authors:** Chaonan Zhang, Rongmao Qiu, Yongjie Huang, Yinghua Liu, Kui Huang, Suwen Zhao, Yang Li

**Affiliations:** ^1^ Guangdong Provincial Engineering Research Center of Molecular Imaging Guangdong‐Hong Kong‐Macao University Joint Laboratory of Interventional Medicine the Fifth Affiliated Hospital Sun Yat‐sen University Zhuhai Guangdong 519000 China; ^2^ Present address: School of Pharmacy and Medical Technology Key Laboratory of Pharmaceutical Analysis and Laboratory Medicine of Fujian Province Putian University Putian Fujian 351100 China

**Keywords:** electrostatic interactions, glycosaminoglycan, peptidomimetics, peptoid residue, serum stability, whole‐body imaging

## Abstract

Although cartilage damage is key to the pathogenesis of many musculoskeletal diseases, including osteoarthritis and rheumatoid arthritis (RA), imaging and drug delivery to cartilage remain a demanding challenge. Cartilage is an avascular tissue with a dense matrix constraining the penetration of imaging and targeting agents. Here, a unique class of cationic peptidomimetics featuring peptoid residue Nlys (N‐substituted butyl‐amino glycine) is reported for cartilage targeting. Sulfo‐Cyanine5 (Cy5) labeled Nlys‐rich sequences are found to penetrate and be retained within millimeter‐deep cartilage plugs in vitro by specific binding to the tissue's polyanionic glycosaminoglycan (GAG) chains. Owing to the unnatural sequences being undegradable by common proteases, these Nlys‐peptidomimetics overcome the problem of low serum stability of existing benchmark cartilage‐binding peptides (e.g., octa‐arginine) and allow systemic administration. Intravenously delivered Cy5‐(NlysO)_7_ (O: hydroxyproline) is taken up by the cartilage across the body of living mice and zebrafish, enabling whole‐body 3D light‐sheet microscopy scans of neonatal mice undergoing bone development as well as in vivo detection of GAG loss in mice's aged knee joints and inflamed RA ankles. The Nlys‐compounds also show no cytotoxicity in chondrocytes and good biocompatibility in vivo, paving the way for applications in GAG‐targeted molecular imaging, drug delivery, and biomaterials for cartilage‐related diseases.

## Graphical abstract

Systemically‐dosed Cy5‐labeled cationic peptoid probe (NlysO)_7_ (yellow) binds to the cartilage's glycosaminoglycan content in vivo across the entire body of a neonatal mouse imaged by light sheet fluorescence microscopy, revealing fluorescence uptake in generic cartilage as well as the developing and ossifying bones.

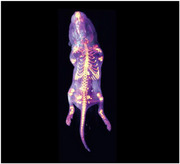

## 1 Introduction

Cartilage performs key structural and mechanical functions for various organ systems in the human body, such as cushioning joints and intervertebral spaces, shaping ears and nose, and maintaining the tracheal structure.^[^ ^1^ ^]^ Structural damage and dysfunction of the cartilage, a tissue that can hardly self‐repair, is a core pathological change in musculoskeletal degenerative diseases (e.g., knee and facet joint osteoarthritis^[^ ^2^ ^]^) impacting the health and quality of life of the aging populations worldwide, and a key factor in disability caused by autoimmune disorders such as rheumatoid arthritis (RA)^[^ ^3^ ^]^ and ankylosing spondylitis.^[^ ^4^ ^]^ However, sensitive radiological detection of cartilage damage is still lacking. Conventional X‐ray radiography cannot visualize the cartilage, so doctors have to rely on bone spacing to infer the extent of cartilage damage, and arthritic joints with less than six months of the disease may appear normal on X‐ray.^[^ ^5^ ^]^ Meanwhile, cartilage is an avascular tissue with an increasingly dense extracellular matrix (ECM) in the deep layers, constraining compound diffusion and penetration. These physiological and physical barriers make it challenging for drugs to reach the cartilage lesions, thereby limiting their efficacy.^[^ ^6^ ^]^ Therefore, targeting molecules with specific affinity for cartilage tissue can become vital tools for new breakthroughs in the diagnosis and treatment of cartilage diseases.

**Figure 1:**
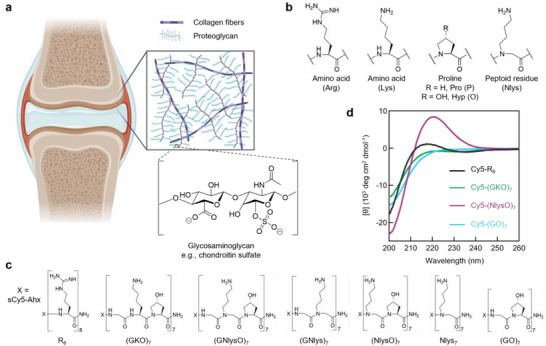
Glycosaminoglycan‐binding peptoids. a) The articular surface of the bone in synovial joints is protected with a layer of hyaline cartilage, whose unique ECM is rich in collagen II and proteoglycans consisting of anionic glycosaminoglycan (GAG) chains. b) An α‐amino acid [e.g., arginine (Arg, R) or lysine (Lys, K)], an imino acid [e.g., proline (Pro, P) or hydroxyproline (Hyp, O)], and a peptoid residue (i.e., N‐substituted glycine, e.g., Nlys) share a common backbone structure, but their sidechains are attached to different atoms. c) Chemical structures of the Cy5‐labeled peptidomimetic compounds studied in this work (Ahx: aminohexanoic acid). d) Circular dichroism (CD) spectra of Cy5‐R_8_, Cy5‐(GKO)_7_, Cy5‐(NlysO)_7_, and Cy5‐(GO)_7_ measured at 25 °C (50 µM in PBS buffer).

The dense matrix of cartilage is composed of a network of collagen fibers and anionic proteoglycans and glycosaminoglycans (GAGs, **Figure** **1**a).^[^ ^7^ ^]^ Exploiting them as targets, several cartilage‐targeting molecules have been reported in recent years. For instance, the chondrocyte membrane was extracted as cartilage‐targeting natural materials.^[^ ^8^ ^]^ Designed and screened synthetic cartilage‐targeting molecules include collagen‐II binding peptide WYRGRL^[^ ^9^ ^]^ as well as GAG‐binding cationic fluorescent dyes,^[^ ^10^ ^]^ peptides,^[^ ^11^ ^]^ and polymers.^[^ ^12^ ^]^ Unfortunately, most of these GAG‐targeting molecules are easily cleared or degraded in circulation due to their large size^[^ ^13^ ^]^ or the instability of their natural sequences^[^ ^14^ ^]^ and have to be delivered locally (i.e., intra‐articular injection);^[^ ^6^, ^11^, ^12^ ^]^ this administration requirement is inconducive to systemic targeting for multisite lesions (e.g., RA or ankylosing spondylitis), which greatly limits their in vivo applications.^[^ ^15^ ^]^

Here we report a class of cartilage‐specific, serum‐stable, cationic peptide‐peptoid compounds for GAG targeting. Peptoids are synthetic peptide‐like polymers built by N‐substituted glycine units. Unlike natural amino acids, peptoid residues have sidechains attached to their amide nitrogen atoms instead of the α‐carbon atoms (Figure 1b),^[^ ^16^ ^]^ and are therefore not recognizable and degradable by common proteases.^[^ ^17^ ^]^ We envision that cationic chains rich in peptoid residues with a moderate molecular size may penetrate the cartilage's matrix barrier and be preferentially taken up by the cartilage. We anticipate such targeting peptidomimetics to have enormous potential applications in molecular imaging, drug delivery, and biomaterials for cartilage‐related diseases.

## 2 Results

### 2.1 Peptoid Design and Characterization

During a previous study of peptoid‐residue‐containing collagen mimetic peptides,^[^ ^18^ ^]^ we stumbled upon a sulfo‐Cyanine5 (Cy5) labeled sequence of (GNlysO)_7_ (G: glycine), which seemingly could be taken up in vivo by the cartilage in normal mice. Here, O stands for (4*R*)‐hydroxyproline; Nlys represents a peptoid residue with an N‐substituted butylamine sidechain, forming an isomer of the amino acid lysine (Figure 1b). We initiated the present study to identify the structural factors underlying the unexpected cartilage affinity of this peptidomimetic compound. We designed and synthesized a few other sequences, including (GKO)_7_ (K: lysine), (NlysO)_7_, (GNlys)_7_, Nlys_7_, (GO)_7_, and R_8_ (R: arginine), and labeled them with a Cy5 fluorophore at the N‐terminus of each sequence through an aminohexanoic acid (Ahx) linker (Figure 1c; Figures S1 and S2, Supporting Information). Here (GKO)_7_ is the natural peptide counterpart of (GNlysO)_7_, where the two differ only in the position of the sidechain‐backbone attachment. In contrast, R_8_ is a widely recognized cationic peptide with known cartilage affinity.^[^ ^11^, ^19^ ^]^ Circular dichroism (CD) spectra of most of these Cy5‐labeled sequences indicated a random coil conformation [e.g., (GKO)_7_, (GO)_7_, (GNlysO)_7_, and R_8_] in the PBS solution (Figure 1d; Figure S1, Supporting Information). Among them, Cy5‐(NlysO)_7_ exhibited a typical CD profile of a polyproline II helix with the highest mean residue ellipticity value at 225 nm (Figure 1d). This structural character may be related to its highest content of imino acids (i.e., Hyp and Nlys residues).^[^ ^18^, ^20^ ^]^ (GNlys)_7_ and Nlys_7_ displayed no structural features at all in CD (Figure S1, Supporting Information), probably due to the lack of a chirality center in the entire molecules.

### 2.2 The Cartilage Affinity

**Figure 2:**
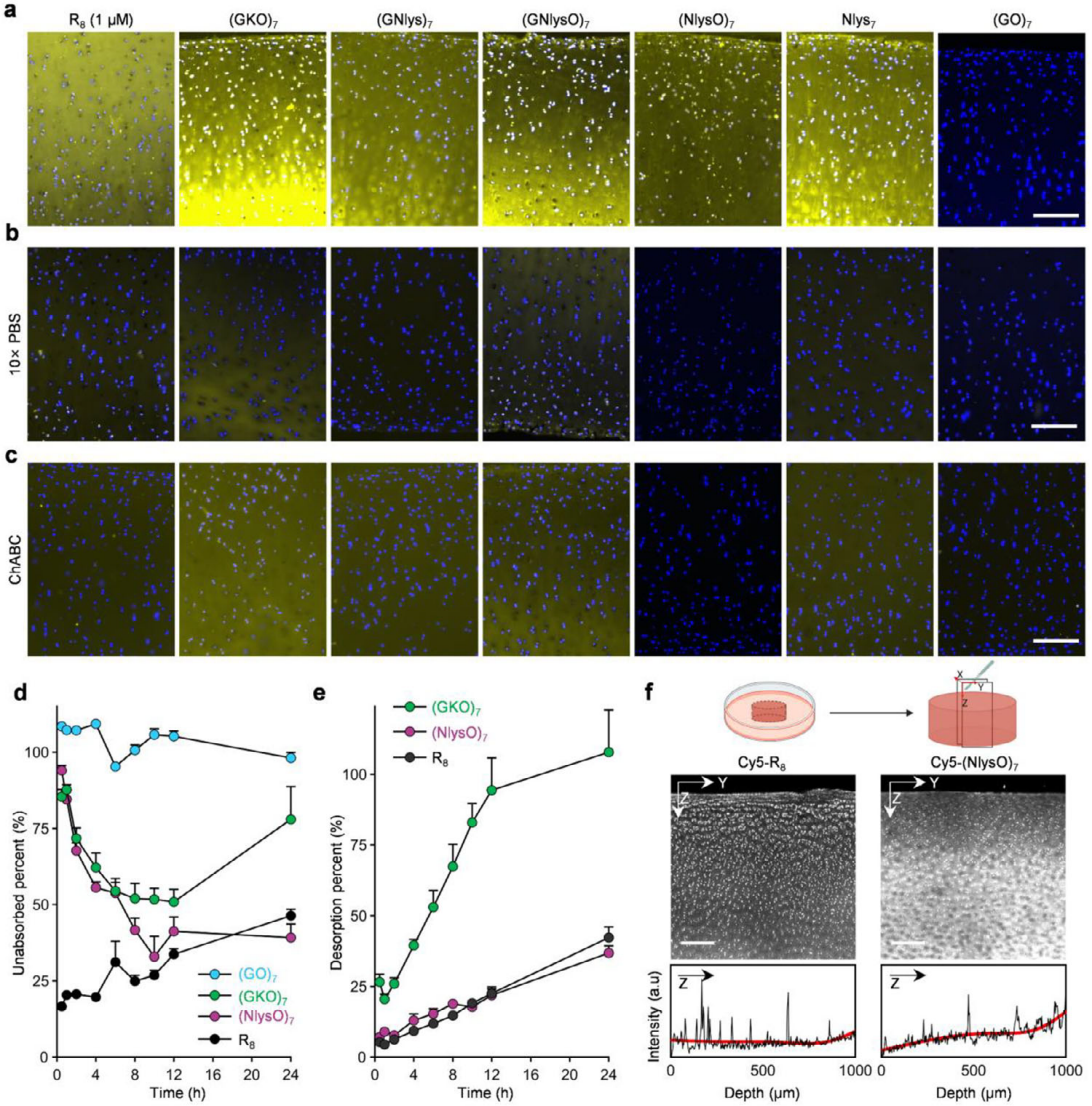
Cartilage cryosection staining and in vitro cartilage uptake, retention, and penetration assays. a–c) Representative fluorescence micrographs of adjacent cryosections of normal porcine cartilage stained with 1 µM of Cy5‐R_8_ or 10 µM of Cy5‐(GKO)_7_, Cy5‐(GNlys)_7_, Cy5‐(GNlysO)_7_, Cy5‐(NlysO)_7_, Cy5‐(Nlys)_7_, or Cy5‐(GO)_7_ and washed with three rounds of 1× PBS (a) or 10× PBS buffer (b). The sections in (c) were pre‐treated with Chondroitinase ABC (ChABC) for 24 h to selectively reduce the GAG content in the cartilage tissue. d,e) The fluorescence intensity changes of the solutions of Cy5‐R_8_, Cy5‐(GKO)_7_, Cy5‐(GO)_7_, or Cy5‐(NlysO)_7_ (starting concentration: 10 µM) over 24 h of incubation at room temperature with porcine cartilage slices (d). Following the absorption experiment in d, the cartilage samples were incubated at room temperature in blank PBS solutions at room temperature, whose fluorescence intensity changes were measured over 24 h (e) [*n* = 6 samples, except two excluded outliers exceeding 100% for Cy5‐(GKO)_7_ in e]. Data: mean + s.e.m. f) Representative confocal microscopy images and spatial fluorescence quantification showing the penetration profiles of Cy5‐R_8_ and Cy5‐(NlysO)_7_ into cylinder‐shaped porcine cartilage plugs (diameter: 6 mm, thickness: 1 mm) that had been soaked in the probe solutions for 24 h. Slices (X: 100‐200 µm, Y: 6 mm, Z: 1 mm) in the center of the plugs were cut out and scanned in the Y‐Z plane by confocal microscopy (*n* = 5 plugs). Scale bars: 150 µm (a‐c), 200 µm (e).

We stained, rinsed, and fluorescently imaged adjacent cryosections of the tibial cartilage of a porcine knee joint using these Cy5‐labeled peptidomimetic probes. (GKO)_7_, (GNlysO)_7_, (NlysO)_7_, (GNlys)_7_, and Nlys_7_ all bound to the cartilage tissue notably and displayed similar fluorescence intensities (**Figure** **2**a). The fluorescence signal also often became progressively stronger away from the cartilage surface, matching the increasing GAG density along the depth into the tissue.^[^ ^21^ ^]^ Interestingly, the cartilage binding of R_8_ appeared to be far stronger than other sequences: with only 1 µM, Cy5‐R_8_ showed a level of fluorescence close to that of the other compounds at 10 times the concentration (10 µM, Figure 2a). The higher in vitro cartilage uptake of R_8_ may be related to i) the stronger cationic nature of its guanidine sidechain compared to that of Nlys^[^ ^22^ ^]^ and ii) its lower molecular weights compared to all other cationic peptidomimetic compounds except Nlys_7_.

Opposite to these cationic sequences, (GO)_7_ showed no cartilage affinity (Figure 2a), suggesting that electrostatic interactions of the probes’ cationic sidechains with the anions in the cartilage matrix are a probable driving force for the cartilage affinity. When we washed the stained cartilage sections with a solution of higher ionic strength (i.e., 10× PBS buffer), the fluorescence intensity of the tissue decreased significantly (Figure 2b). This result further supports our hypothesis that electrostatic attraction mediates the affinity since the electrostatic effects are weakened in solutions with high ionic strength.^[^ ^23^ ^]^ Next, we found that all these cationic sequences lost much of their cartilage affinity after we treated the tissue sections with Chondroitinase ABC (ChABC) to selectively remove their GAG component (Figure 2c).^[^ ^24^ ^]^

The pKa values of the sidechain amino and guanidine groups in Lys and Arg are 10.7 and 13.8, respectively,^[^ ^22^ ^]^ indicating that the basic sidechain groups in our peptidomimetics should be positively charged at the physiological pH. This was supported by the ^1^H NMR spectra of the cationic sequences before and after alkalization of their solutions (Figure S3, Supporting Information). We also measured the zeta potential of the chondroitin sulfate (CS) biopolymer chains, the primary GAG type in the cartilage, in water with or without the addition of the probes. As expected, the zeta potential value of CS solution rose extensively from ‐38 mV to ≈ ‐10 mV with the addition of the cationic Cy5‐(GNlys)_7_, Cy5‐(NlysO)_7_, Cy5‐Nlys_7_, Cy5‐(GKO)_7_, and Cy5‐(GNlysO)_7_, and even to +3 mV with Cy5‐R_8_, but not with the neutral Cy5‐(GO)_7_ (Figure S4, Supporting Information). Together, these results directly demonstrate that these cationic peptidomimetic molecules bind to the cartilage's GAGs via electrostatic interactions.

### 2.3 In Vitro Cartilage Uptake, Retention, and Penetration

We examined the cartilage uptake, retention, and penetration of the peptidomimetic probes. A 10 mg slice of porcine cartilage was incubated with a probe (starting concentration: 10 µM), and the remaining fluorescence intensity in the solution was measured regularly over 24 h (Figure 2d). We found that Cy5‐R_8_ was taken up very rapidly from the solution by the cartilage, with only ≈ 20% of the starting concentration remaining after 30 min. In contrast, the concentration of the (GO)_7_ solution remained almost constant due to the absence of cartilage affinity. The concentration of the unabsorbed (NlysO)_7_ decreased progressively over the first 10 h and became equilibrated at 24 h (Figure 2d); other Nlys‐containing compounds followed a similar trend (Figure S5, Supporting Information). Interestingly, the Cy5 concentrations in the supernatants of R_8_ and (GKO)_7_ first decreased quickly and then gradually increased again (Figure 2d), suggesting that the two peptides may not be firmly retained in the tissue after the initial binding or could be digested by the remaining proteases in the tissue.^[^ ^25^ ^]^

After 24 h of probe absorption, we incubated the cartilage slices in 200 µL of blank PBS solution at room temperature and monitored the desorption of the cationic compounds by measuring the Cy5 fluorescence intensity in the supernatants. The dissociation rate of (NlysO)_7_ was similar to R_8_ but drastically lower than that of (GKO)_7_ (Figure 2e). All Nlys‐containing probes showed stable cartilage retention, but the desorption of (GNlys)_7_ and Nlys_7_ was slightly slower (Figure S6, Supporting Information).

To visually assess the probes’ cartilage penetration, we added a solution of Cy5‐R_8_ or Cy5‐(NlysO)_7_ on top of a 1‐mm‐high porcine cartilage plug (diameter: 6 mm) for 24 h incubation at room temperature. Afterward, confocal microscopy imaging of the central cross‐section of these cartilage samples showed that Cy5‐(NlysO)_7_ had penetrated to the bottom of the 1‐mm‐deep tissue and distributed in the cartilage matrix with its fluorescence signal gradually increasing along the tissue depth (Figure 2f). In contrast, the fluorescence distribution of R_8_ did not show this consistency with the GAG density but appeared more co‐localized with the chondrocytes (Figure 2f), possibly related to R_8_’s additional ability to penetrate the cell membrane.^[^ ^26^ ^]^ To validate this hypothesis, we performed the cellular uptake assays of these cationic compounds on rat chondrocytes. Indeed, we noted strong uptake of Cy5‐R_8_ and some weak uptake of Cy5‐Nlys_7_ compared to the negligible amounts of Cy5‐(GNlys)_7_, Cy5‐(GNlysO)_7_, Cy5‐(GO)_7_, Cy5‐(GKO)_7_, and Cy5‐(NlysO)_7_ taken up by the chondrocytes from the culture medium over 24 h (Figure S7, Supporting Information).

Together, these results revealed the strong penetration of the Nlys‐containing peptidomimetics and their stable GAG binding and retention in the cartilage matrix.

### 2.4 Serum Stability

**Figure 3:**
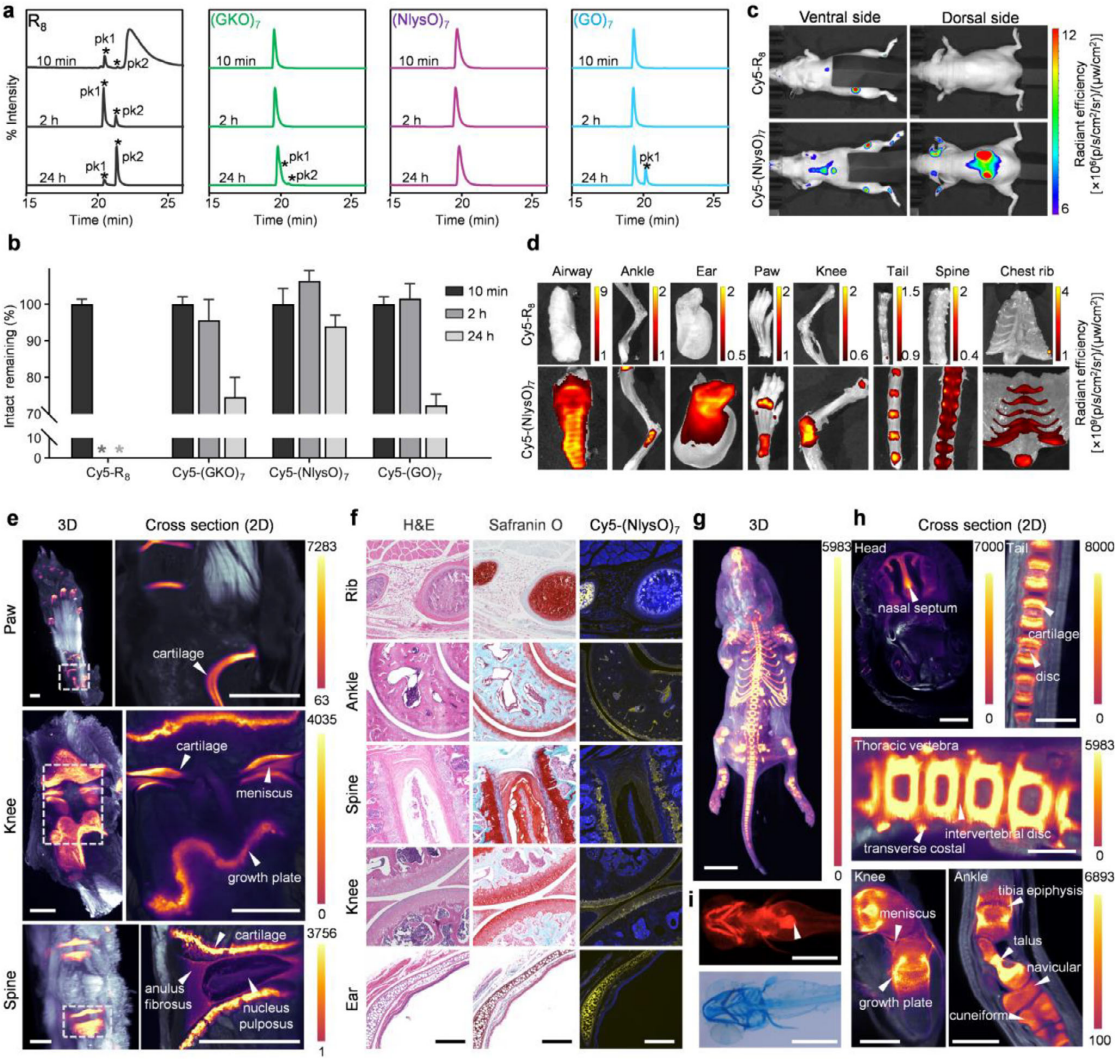
Serum stability of Cy5‐R_8_, Cy5‐(GKO)_7_, Cy5‐(NlysO)_7_, and Cy5‐(GO)_7_ and in vivo cartilage targeting of Cy5‐(NlysO)_7_. a) Representative HPLC traces of Cy5‐R_8_, Cy5‐(GKO)_7_, Cy5‐(NlysO)_7_, and Cy5‐(GO)_7_ after they were incubated in 25% v/v mouse serum for 10 min, 2 h, or 24 h at 37 °C. (UV detection: 646 nm; *: fragment peak). b) Histograms showing the percent of remaining intact compounds for Cy5‐R_8_, Cy5‐(GKO)_7_, Cy5‐(NlysO)_7_, and Cy5‐(GO)_7_ after they were incubated in mouse serum for 10 min, 2 h, and 24 h, calculated from integrated peak areas in HPLC (*: undetected). Data: mean + s.e.m. (*n* = 3 samples). c) In vivo fluorescence images of 16‐week‐old nude mice 2 h post tail‐vein injection of 1 nmol of Cy5‐R_8_ or Cy5‐(NlysO)_7_ (*n* = 4 mice). d) Representative fluorescence images of the cartilage‐rich tissues collected from the mice in c 2 h post‐injection of Cy5‐R_8_ or Cy5‐(NlysO)_7_. e) Light‐sheet fluorescence microscopy images of the front paw, knee joint, and spine specimens collected from c and d showing the specific anatomic locations of Cy5‐(NlysO)_7_′s in vivo cartilage uptake. f) Representative micrographs of adjacent paraffin‐embedded sections of the cartilage tissues from the mice intravenously injected with Cy5‐(NlysO)_7._ For each tissue sample, three adjacent section slides were prepared and imaged after deparaffinization: one stained with H&E (left), another one stained with Safranin O (middle), and the last one (right) counterstained with DAPI (blue) and fluorescently imaged to visualize the anatomic location of the in vivo bound Cy5‐(NlysO)_7_ (yellow). g,h) Light‐sheet fluorescence microscopy image of the whole body (g, 3D) and views of local anatomic structures (h, 2D) of a 1‐week‐old neonatal mouse post intravenous injection of Cy5‐(NlysO)_7_. i) Representative fluorescence images of live zebrafish 5 days post‐fertilization (dpf) 2 h post‐injection of 0.1 pmol of Cy5‐(NlysO)_7_ (arrowhead: injection site) (*n* = 6 fish) as well as a micrograph of a fixed zebrafish (5 dpf) stained by Alcian blue (bottom). Scale bars: 1 mm (e, h), 275 µm (f), 5 mm (g), 500 µm (i).

We examined the stability of the cationic probes after incubating them with 25% mouse serum using high‐performance liquid chromatography (HPLC). We found that R_8_ was extremely unstable: after mixing with serum for even less than 10 min (the peptide was isolated by centrifuge immediately after addition to serum; deadtime ≤ 10 min), the R_8_ samples displayed fragment peaks in HPLC that could be identified by MALDI‐MS (pk 1 and 2, **Figure** **3**a; Figure S8e, Supporting Information); no intact R_8_ peptide remained after 2 h incubation (Figure 3a,b). These results agree with previous reports on the serum instability of R_8_.^[^ ^27^ ^]^ Meanwhile, (GO)_7_ and (GKO)_7_ also had ≈ 25% of the peptides proteolyzed after 24 h of serum incubation (Figure 3a,b; Figure S8, Supporting Information). In contrast, (NlysO)_7_, (GNlysO)_7_, and (GNlys)_7_ were hardly degraded in the serum (Figure 3a,b; Figure S8, Supporting Information) even after 48 h (Figure S9, Supporting Information). Even though these peptidomimetic compounds are also highly cationic, they differ from R_8_ and (GKO)_7_ in that their sequences are rich in unnatural peptoid residue Nlys and noncanonical imino acid Hyp, both of which may protect them from recognition and degradation by serum proteases.^[^ ^28^ ^]^

### 2.5 Systemic In Vivo Cartilage Targeting

The high serum stability of these cationic Nlys‐containing sequences prompted us to test whether the cartilage can be targeted and fluorescently imaged in vivo via systemic administration of these probes.

We first monitored the dynamic changes of the in vivo fluorescence signals in nude mice over 96 h post‐tail‐vein injection of 1 nmol of each probe (Figure S10, Supporting Information). We found that the fluorescence signals of the Nlys‐rich peptidomimetics in the knee joints and thoracic costal cartilage region were stronger than those with Cy5‐R_8_ and Cy5‐(GKO)_7_ at each time point and remained detectable 8–12 h post‐injection, whereas the signals from Cy5‐R_8_ were difficult to find after 2 h post‐injection (Figure S10, Supporting Information). These in vivo observations were consistent with our in vitro serum stability experiments (Figure 3a,b). Observations and signal quantifications of the fluorescence images of individual live mice (Figure S10, Supporting Information), as well as harvested skeletal and cartilage tissues (Figures S11 and S12, Supporting Information), all suggested that with a 1‐nmol dose, these Nlys‐compounds can be retained in the cartilage for as long as 48 h and be almost fully removed 96 h post‐injection. Fluorescence imaging of major organs dissected 2 h post‐injection showed that the cationic peptoid probes were mainly cleared through the kidneys and (modestly) liver (Figure S13, Supporting Information). Fluorescence imaging of nude mice obtained 2 h post‐tail‐vein injection showed in vivo accumulation of fluorescence signals for the mice dosed with Cy5‐(NlysO)_7_ but not Cy5‐R_8_ in the cartilaginous tissues, including the knee joints, ears, noses, ribs, spines, and ankles (Figure 3c; Figure S14, Supporting Information). Close‐up examination of the dissected tissues of the Cy5‐(NlysO)_7_ dosed mice indicated that the entire cartilage anatomy was fluorescently labeled, including the trachea, ears, paws, knee joints, tails, spines, and costal cartilage (Figure 3d; Figure S15, Supporting Information).

Three‐dimensional light‐sheet fluorescence microscopy of the dissected and cleared tissue specimens distinctively revealed the in vivo uptake of Cy5‐(NlysO)_7_ in the interphalangeal tuberosity in the front paws, in the articular cartilage, the growth plates, and the meniscus of the knee joints, as well as in the endplates (and weakly in the intervertebral discs) of the spine (Figure 3e). The in vivo fluorescence cartilage uptake could even be preserved in the paraffin‐embedded tissue sections, and matched perfectly with the GAG content revealed by the Safranin O stain of adjacent sections (Figure 3f). These images and histology results demonstrate that the cationic peptidomimetic (NlysO)_7_ can target all three types of cartilage in vivo (e.g., hyaline cartilage: articular cartilage, fibrocartilage: meniscus, elastic cartilage: ear).

Using these methods, we found that Cy5‐(NlysO)_7_ accumulated in the cartilage throughout the body of 1‐week‐old neonatal mice in vivo (Figure 3g; Figure S16, Supporting Information): in addition to generic cartilage tissues (e.g., nasal septum), we noted even stronger probe uptake in the deteriorating cartilage matrix of the developing and ossifying bones, including the thoracic and tail vertebrae, the transverse costal, as well as the active and thickened epiphyses of the knee and ankle joints (Figure 3h; Video S1, Supporting Information). Furthermore, we discovered that the developing skull cartilage of live zebrafish, including the pharyngeal arches and the cerebral‐cranial cartilage, can be vividly marked in vivo by the Cy5‐(NlysO)_7_ probe injected into the hemolymph cavity 5 days post‐fertilization (Figure 3i; Video S2, Supporting Information). These (NlysO)_7_‐labeled anatomical structures are consistent with the Alcian blue stain results (Figure 3i). Together, these findings showcase the potential in vivo applications of cationic cartilage‐binding peptidomimetics for probing musculoskeletal development in various model animals.

### 2.6 Visualizing Cartilage Degeneration and Erosion

**Figure 4:**
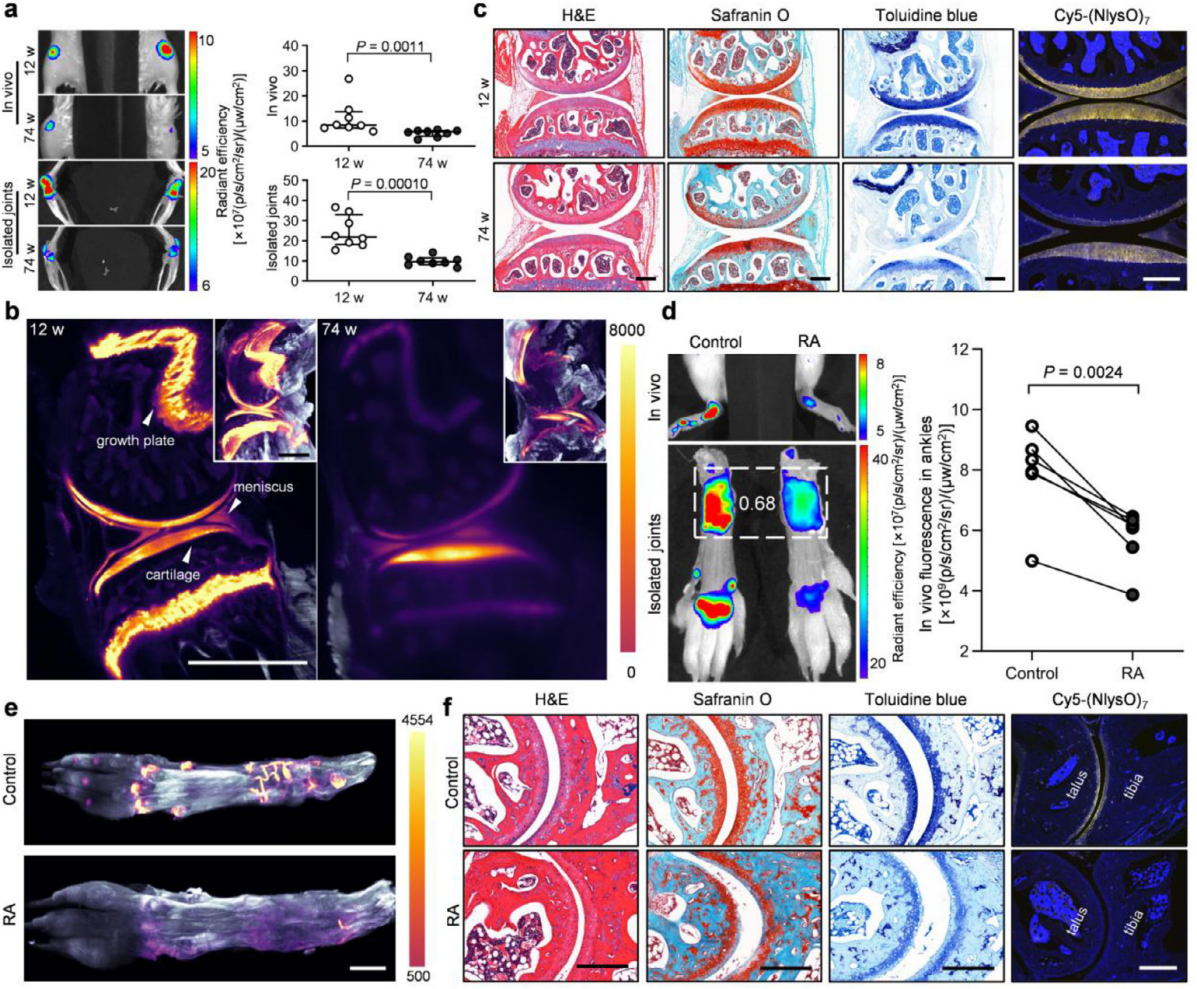
Detecting GAG loss in aging knees and inflamed ankles in vivo by Cy5‐(NlysO)_7_. a) Representative fluorescence images (in vivo and isolated joints) and region‐of‐interest signal quantification for the knee joints in young (12‐week‐old) and old (74‐week‐old) Balb/c mice acquired 2 h post‐intravenous injection of 2 nmol Cy5‐(NlysO)_7_. Data (*n* = 4 mice, points represent individual paws): median with interquartile range. Statistics: Mann‐Whitney U test (in vivo) or Welch's t‐test (isolated joints). b) Light‐sheet fluorescence microscopy images [sagittal cross‐section and 3D (top right thumbnails)] of the knee joints from the mice studied in (a) showing strongly decreased Cy5‐(NlysO)_7_ uptake in the articular cartilage and growth plates of the old mice compared to that of the young mice. c) Histologic stains (H&E, Safranin O, Toluidine blue) of paraffin‐embedded sections of the knee joints from the mice studied in (a) and in situ fluorescence from the tissue‐bound Cy5‐(NlysO)_7_ in another adjacent section (yellow, blue: DAPI). d) Representative fluorescence images (in vivo and isolated paws) and region‐of‐interest signal quantification for the ankle joints in the arthritic hind paw compared to its non‐arthritic control in single‐paw collagen‐antibody‐induced‐arthritis (CAIA) model mice, acquired 2 h post‐intravenous injection of 2 nmol Cy5‐(NlysO)_7_. The ratio of quantified Cy5‐(NlysO)_7_ fluorescence signals from the ankle regions between the arthritic and the control hind paws of the mouse is shown inside the dashed box (*n* = 6 mice, paired t‐test). e) Light‐sheet fluorescence microscopy scanned 3D images of the hind paws of a single‐paw CAIA model mouse pre‐injected with Cy5‐(NlysO)_7_. f) Histologic stains (H&E, Safranin O, Toluidine blue) of paraffin‐embedded sections of the ankles from the mice studied in (d) and in situ fluorescence from the tissue‐bound Cy5‐(NlysO)_7_ in another adjacent section (yellow, blue: DAPI). Scale bars: 1000 µm (b); 275 µm (c, f); 2000 µm (e).

GAG loss is a hallmark and contributing factor in cartilage aging and disease. For instance, GAG degradation mediated by ADAMTS (a disintegrin and metalloproteinase with thrombospondin motifs) proteases is a main driver of cartilage degeneration in osteoarthritis and a key cartilage destructing event during RA.^[^ ^29^ ^]^ Therefore, toward the goal of developing a molecular imaging tool for assessing GAG loss in the cartilage noninvasively, we attempted to differentiate the degenerated or inflamed joints from the normal ones by in vivo fluorescence imaging with Cy5‐(NlysO)_7_ in mice (**Figure** **4**). Two hours post‐intravenous injection, the Cy5‐(NlysO)_7_ uptake in the knee joints of the aged mice (74‐week‐old) was drastically lower than that of the young mice (12‐week‐old) (Figure 4a; Figure S17, Supporting Information) with the median fluorescence signals from the knee regions only half that of the young controls (Figure 4a). Light‐sheet fluorescence microscopy scan of the cleared joint specimens from these mice suggested that the probe's binding in the aged mice's articular cartilage and, more prominently, the growth plates was notably lower than that of the young mice (Figure 4b). The low fluorescence from the Cy5‐(NlysO)_7_ probe retained in the paraffin‐embedded sections of the dissected joints also indicated significant GAG loss from the aged mice's degenerating articular cartilage and meniscus, a result corroborated by the Safranin O and Toluidine blue stains of the adjacent sections (Figure 4c).

For RA imaging, we injected Cy5‐(NlysO)_7_ into the tail veins of a group of model mice, where arthritis was locally triggered in their left hind paws.^[^ ^30^ ^]^ The in vivo fluorescence imaging, as well as the postmortem light‐sheet microscopy scanning and histology analysis of the joints, all showed that the Cy5‐(NlysO)_7_ uptake in the cartilage of the arthritic ankle was significantly lower than that of its non‐arthritic counterpart in each mouse (Figure 4d–f; Figure S18, Supporting Information). These findings strongly showcase the possibility of examining GAG loss in joint degeneration and arthritis non‐invasively via imaging instead of relying on endpoint histology.

Many polycationic compounds are known to permeate cell membranes and cause cell death.^[^ ^31^ ^]^ Our cytotoxicity stains (live/dead fluorescence, Figure S19, Supporting Information) and cell viability tests (CCK8 assays, Figure S20, Supporting Information) both indicated that, unlike R_8_, these peptoid‐based cationic sequences were not toxic to the cultured chondrocytes. Moreover, under the tested concentrations, Cy5‐(NlysO)_7_ did not show cytotoxicity to the chondrocytes over 72 h (Figure S21, Supporting Information); it also did not exhibit cytotoxicity to cultured human umbilical vein endothelial cells (HUVECs, Figure S22, Supporting Information). In contrast, Cy5‐R_8_ was readily taken from the culture media by both cells (Figures S7 and S22c, Supporting Information), leading to significant cytotoxicity to them (Figures S20f and S22a, Supporting Information). Furthermore, the biochemical analysis of the blood collected from the mice 24 h post intravenous injection of 2 nmol Cy5‐(NlysO)_7_ showed that no key analytes from the Cy5‐(NlysO)_7_ group significantly differed from those that only received PBS buffer (Figure S23, Supporting Information). Routine blood test results of normal mice with three repeated weekly injections of the Nlys‐probes also did not differ from those of the control ones dosed with PBS buffer (Figure S24, Supporting Information). No hemorrhagic, inflammatory, or other pathologic indications were identified through histology analysis of the major organs from mice injected with the Nlys‐containing probes (Figure S25, Supporting Information). Together, these data provide a preliminary manifestation of the biocompatibility of these peptoid‐based cationic cartilage‐targeting agents, while the chronic toxicity and immunogenicity remain to be monitored in future research.

Finally, we prepared biotin‐(NlysO)_7_ as a preliminary model of a Nlys‐peptidomimetic drug conjugate, with biotin representing a small‐molecule drug (Figure S26, Supporting Information). We intravenously injected this conjugate into normal mice and collected their joint tissues (Figure S26b–d, Supporting Information). Following fluorescence staining of the isolated femur cartilage with Cy3‐labeled streptavidin, we demonstrated the accumulation of the biotin moiety in the cartilage from the mice dosed with the biotin‐(NlysO)_7_ conjugate (Figure S26e, Supporting Information). These observations suggest that Nlys‐peptidomimetics warrant further development as future drug carriers to the cartilage.

## 3 Discussion

Since the 1990s, peptoids (i.e., polymers and oligomers of N‐substituted glycines) have been developed in many biomedical fields as synthetic compounds that mimic the structure and function of natural proteins,^[^ ^32^ ^]^ particularly as antimicrobial compounds^[^ ^33^ ^]^ as well as targeting and imaging agents for cell receptors (e.g., tumor receptors,^[^ ^34^ ^]^ VEGFR^[^ ^35^ ^]^ and neurotensin receptors^[^ ^36^ ^]^). Nonetheless, there are no reports about peptoids for cartilage targeting and imaging to date. As an avascular tissue, the cartilage is difficult to engage by molecular agents from the bloodstream, usually requiring local intra‐articular delivery.^[^ ^11^, ^12^ ^]^ Cartilage binding molecules are typically highly cationic and easily degraded and metabolized in the serum before reaching the cartilage (e.g., R_8_ as shown in Figures 2d,e and 3a,b; Figure S8e, Supporting Information). Common serum proteases and peptidases include carboxypeptidase N (cleavage sites: Ala, Leu, Arg, Trp, Leu, and Phe), aminopeptidase N (cleavage sites: Ala, Leu, Arg, Trp, Leu, and Phe), plasmin (cleavage sites: Arg and Lys), and furin (cleavage sites: Arg, Ser, and Lys);^[^ ^37^ ^]^ they can easily degrade peptides rich in Lys or Arg, making most cartilage‐binding cationic peptides and polymers unstable in serum and less suitable for systemic delivery.^[^ ^12c,d^ ^]^ The unnatural N‐substituted peptoid residues, featuring a bulky sidechain attached to the backbone's amide nitrogen, provide a chemical strategy to improve a cationic probe's serum stability by rendering the amide sites unrecognizable by the common serum proteases.^[^ ^17^ ^]^

As a proof‐of‐concept, our study demonstrates that the high serum stability of the peptoid and hydroxyproline‐rich sequences against protease degradation (Figure 3a; Figures S8 and S9, Supporting Information) can offer these cationic peptidomimetics [i.e., (Nlys)_7_, (GNlys)_7_, (GNlysO)_7_, and (NlysO)_7_] the advantage of a wider metabolic time window in vivo (Figures S10–S12, Supporting Information) compared to generic peptides, enabling sufficient cartilage delivery (Figure 3d–h). Additionally, their small sizes and low molecular weights (Figures S1 and S2, Supporting Information) may also be conducive to penetrating the physical barrier of the cartilage matrix. Importantly, these Nlys probes should be positively charged under physiological conditions (Figure S3, Supporting Information), enabling direct electrostatic interactions with the highly negatively charged GAGs (e.g., chondroitin sulfate) in the cartilage (Figure S4, Supporting Information). Together, our peptidomimetic findings underline the positive charges and in vivo stability^[^ ^15^ ^]^ as the basic design principles for cartilage‐targeting molecules. Although their conformations can be quite distinct as shown by CD (Figure S1, Supporting Information), our Nlys‐sequences all share these two properties (Figures S3–S9, Supporting Information) and allow in vivo cartilage targeting (Figures S10–S12, Supporting Information). Future screening and investigation of the structural‐function relationships may reveal more insights into the optimal backbone structure and sidechain functional groups required for high‐affinity GAG‐binding peptoids.

As a sidenote, we found that compared to the cartilage, there was virtually no in vivo fluorescence uptake of Cy5‐(NlysO)_7_ in the mice's vitreous bodies and skin (Figure S27, Supporting Information), despite their high hyaluronic acid contents. We speculate that the cartilage's overall super‐high negative charge density may partially explain the in vivo differences. At the molecular level, the selectivity of the Nlys cationic probes against natural polysaccharides with varying negatively charged units, including chondroitin sulfate and hyaluronic acid (Figure S27, Supporting Information), may require further in vitro biophysical investigation. Meanwhile, the exact mechanism by which our Nlys probes traverse the vascular endothelial barrier to reach the cartilage in vivo requires further exploration, even though peptides/peptoids with similar sizes have been reported to reach their target tissues in vivo from circulation following intravenous administration.^[^ ^15^, ^35^, ^36^ ^]^ We speculate that our Nlys probes may cross the vascular endothelium thanks to their small sizes (≈600‐1000 Da without the Cy5‐labeling, still comparable to small‐molecule drugs). They may also adhere transiently to the weakly anionic albumin in the serum, reaching the joints in the synovial fluids with the albumin, then gradually infiltrating the cartilage matrix to bind tightly with the highly anionic GAGs.

Intra‐articular injection is the main route for delivering drugs to the cartilage in clinical or pre‐clinical procedures.^[^ ^13^ ^]^ However, most drugs are rapidly cleared from the joint space through the lymphatic system and capillaries; for instance, the average half‐life of nonsteroidal anti‐inflammatory drugs (NSAIDs) in the synovial fluid is only 1‐4 h.^[^ ^13^, ^38^ ^]^ This short duration limits the overall therapeutic efficacy. Moreover, joint diseases often cause bone spurs and synovial fluid accumulation, leading to joint space narrowing^[^ ^39^ ^]^ and making intra‐articular injection challenging. Furthermore, compared to intravenous administration, intra‐articular injection is more invasive and more prone to infection;^[^ ^40^ ^]^ it may also be tricky to ensure equal dosage to both knee joints of an individual subject in small animal experiments. More importantly, many non‐articular cartilage structures cannot be accessed through intra‐articular dosing, such as the trachea, ribs, and ears. Our study shows that systemically administered cationic peptoid probes, such as Cy5‐(NlysO)_7_, can lead to fluorescent labeling of different types of cartilage in various anatomical structures throughout the body of zebrafish and mice (Figure 3). (It remains to be tested if they recognize the newly discovered lipocartilage.^[^ ^41^ ^]^) Because of their exceptional protease stability, we also anticipate that these fluorescent Nlys probes can penetrate and remain in the cartilage in vivo with a largely intact chemical structure; this notion is further supported by the positive biotin detection in the cartilage from mice dosed with the biotin‐(NlysO)_7_ conjugate (Figure S26, Supporting Information). This is almost impossible for common GAG‐binding peptide agents, such as R_8_, which is typically delivered intra‐articularly.^[^ ^11a^ ^]^ These findings will lead to unprecedented tools for in vivo monitoring and evaluation of the development (e.g., endochondral ossification), aging, and injury (e.g., bone fracture and healing) of the musculoskeletal system.

The stable negative charges of the GAG chains, providing the normal articular cartilage with the necessary hydration, expansion pressure, and compressive resistance, are key to the mechanical function of the tissue.^[^ ^21^ ^]^ The reduction of GAG during arthritis decreases the cartilage compressive stiffness and lubrication capacity.^[^ ^42^ ^]^ The loss of GAG in the cartilage has been widely accepted as a hallmark of early joint degeneration and arthritis.^[^ ^43^ ^]^ However, Safranin O stain, the routine histological technique for assessing the cartilage's GAG levels, is difficult to quantify, rendering it less ideal to measure any subtle changes in GAG content. In contrast, in our tests, the peptoid probes we developed, such as Cy5‐(NlysO)_7_, can accurately reflect the GAG content changes in the cartilage tissues through histological staining and quantitative fluorescence image analysis (Figures 2, 3, 4; Figure S18b, Supporting Information). More importantly, we show that Cy5‐(NlysO)_7_ can faithfully report the loss of GAG due to age‐related joint degeneration or RA in vivo (Figure 4), which is impossible for Safranin O stain. Our data also demonstrate the biocompatibility of these peptoid probes preliminarily (Figures S19–S25, Supporting Information) and the possibility of carrying bioactive agents to the cartilage with them (Figure S26, Supporting Information). These findings pave the way for the future development and biomedical applications of these GAG‐targeting agents in molecular imaging,^[^ ^44^ ^]^ targeted drug delivery,^[^ ^45^ ^]^ and biomaterials for joint lubrication.^[^ ^46^ ^]^

## 4 Experimental Section

### Solid Phase Synthesis

All peptides or peptoids were synthesized on a Rink Amide‐AM resin using a PurePep Chorus peptide synthesizer via standard Fmoc‐chemistry procedures. Residue Nlys was incorporated on‐resin using the submonomer method.^[^ ^47^ ^]^ See Supplementary Methods in the Supporting Information for details.

### Circular Dichroism (CD) Spectroscopy

The CD spectra were measured using a JASCO J‐1500 CD spectrophotometer. Peptide or peptoid stock solutions were diluted to 50 µM in 1× PBS buffer. The samples were scanned from 200 to 260 nm in a quartz cuvette of 0.1 cm path length at 25 °C. The mean residue ellipticity (MRE, [θ]) was determined using the equation [θ] = (θ × m)/(c × l × n) [θ: measured ellipticity (mdeg); n: the number of residues; m: the molecular weight (g/mol); c: the concentration (mg/mL); l: the path length of the cuvette (mm)]. Each reported CD spectrum was the averaged result of two independent scans after background correction with signals from the blank PBS buffer. CD parameters: bandwidth, 5 nm; digital integration time, 16 s; scanning speed, 20 nm min^−1^; data pitch, 0.1 nm.

### Zeta Potential Measurement

The zeta potentials of the chondroitin sulfate sodium solutions were measured by a Zetasizer Pro dynamic light scattering system (Malvern Panalytical) at room temperature. Each tested peptidomimetic compound (11.6 nmol) was added to a CS solution (0.02 mg mL^−1^ in 1 mL H_2_O) and was mixed for 10 min before measurements.

### Animal Models and Tissues—Mice

All procedures for mouse maintenance and experiments strictly followed the policy of the Experimental Animal Ethics Committee of the Fifth Affiliated Hospital of Sun Yat‐sen University (protocol numbers: 00361, 00436). Balb/c nu mice (male, 10‐18 weeks old) were used for the general in vivo imaging experiments (Figure 3c–f; Figures S10–S14, Supporting Information). For Figure 3g,h and Figure S16 (Supporting Information), neonatal Balb/c mice (7 days old) were used. Female Balb/c mice (74 weeks old versus 12 weeks old) were used in the aged joint imaging experiment reported in Figure 4a–c and Figure S17 (Supporting Information). The RA model was made using female Balb/c mice (6‐8 weeks old) by intraperitoneal injection of an anti‐collagen antibody cocktail (1.5 mg per mouse; Chondrex, 53100) followed by selective subcutaneous injection of lipopolysaccharide (LPS, 12.5 µg per mouse, Chondrex, 53100) into the left hind paw three days later.^[^ ^30^ ^]^ C57BL/6J mice (male, 6–8 weeks old) were used for the biocompatibility tests (Figures S23–S25, Supporting Information).

### Zebrafish

Wild‐type AB zebrafish lines (obtained from the China Zebrafish Resource Center) were maintained under standard conditions (28.5 °C, with 14 h in light and 10 h in dark) in an automatic lighting system (Haisheng). All procedures involving zebrafish strictly followed the policy of the Experimental Animal Ethics Committee of the Fifth Affiliated Hospital of Sun Yat‐sen University (protocol number: Z00005). Zebrafish embryos were collected by natural spawning and kept in an E3 culture medium (5 mm NaCl, 0.17 mm KCl, 0.33 mm CaCl_2_, and 0.33 mm MgSO_4_) with 0.003% N‐phenylthiourea (Sigma, P7629) to reduce melanin deposition.

### Porcine Cartilage Plugs and Sections

Porcine cartilage was freshly harvested from the articular cartilage of an adult pig's knee joint obtained from a local slaughterhouse. Porcine cartilage plugs (diameter: 6 mm) were excised using an osteotomy drill with constant rinsing with PBS buffer for cooling. All solutions in the experiments with unfixed porcine cartilage samples (e.g., Figure 2d–f; Figures S5 and S6, Supporting Information) contained 100 U mL^−1^ penicillin and 0.1 mg mL^−1^ streptomycin (Solarbio, P1400) to prevent bacterial growth.

### Cartilage Cryosection Fluorescence Staining

Porcine cartilage tissue longitudinally cryosectioned from the articular surface to the subchondral bone side into 10 µm‐thick sections was attached to standard glass slides and incubated in PBS solutions containing 10 µM Cy5‐labeled peptidomimetic compounds overnight at 4 °C, followed by washing with 1× PBS buffer and counterstaining with DAPI. To test the binding specificity to GAG, one set of cartilage sections was pre‐treated with Chondroitinase ABC (ChABC; Sigma, C3667, 0.25 U mL^−1^, 200 µL per section) at 37 °C for 24 h to remove the GAG content before probe staining. Following cartilage staining, slides were gently rinsed with 10× PBS buffer at room temperature to test the probes’ binding in solutions with high ionic strength. All sections were imaged using an EVOS M7000 imaging system (Thermo Fisher, Light cubes: Cy5 and DAPI, Objective: 4×). Because the frozen cartilage tissue sections are prone to detachment from the glass slides during washing, we gently rinsed the slides to prevent the detachment. Consequently, we noted that some individual sections (perhaps with a denser matrix than others) in the 10× PBS and ChABC groups may randomly have incomplete fluorescence removal in Figure 2b,c. This result occurred naturally due to a technical limitation and was unrelated to the cartilage‐binding capacities of individual probes.

### In Vitro Cartilage Uptake and Desorption (Retention)

The porcine cartilage plugs were carefully cut into small cuboids with a uniform size (≈5 × 1 × 2 mm, ≈10 mg by wet weight). Each cuboid sample was incubated in a solution of a Cy5‐labeled compound (10 µM in 200 µL 1× PBS buffer per sample) in a 96‐well plate (*n* = 6 samples, one sample per well) at room temperature, and the cartilage samples stayed intact throughout the experiment. Each incubation bath was aliquoted so its fluorescence F_N1_ of the incubation bath was measured without the cartilage samples using a microplate reader (PerkinElmer EnVision; excitation: 646 nm, emission: 662 nm) at specified time points (N1 = 0.5, 1, 2, 4, 6, 8, 10, 12, and 24 h) to estimate the absorption of the probes (Figure 2d; Figure S5, Supporting Information), while fluorescence levels of the compound solutions pre‐measured before incubation were recorded as F_pre_. The unabsorbed percent was calculated as F_N1_/F_pre_×100%, and the final fluorescence measurement after 24 h of absorption was recorded as F_24h_. The desorption study was conducted after the cartilage samples were incubated with the probes for 24 h. Each sample was briefly rinsed with 1× PBS buffer to remove the surface‐bound probe solution and transferred into a well of 200 µL of blank 1× PBS buffer for incubation in a 96‐well plate at room temperature (Figure 2e; Figure S6, Supporting Information). The fluorescence F_N2_ of the washing solution in each well was measured using a microplate reader at designated time points (N2 = 0.5, 1, 2, 4, 6, 8, 10, 12, and 24 h). The desorption percent was calculated as F_N2_/F_absorbed_×100%, where F_absorbed_ is the averaged fluorescence change over the whole absorption experiment for each compound (F_absorbed_ = F_pre‐_F_24h_).

### Confocal Fluorescence Microscopy (Porcine Cartilage Slices and Zebrafish)

Porcine cartilage disks (6 mm in diameter) were incubated in 200 µL of solution of Cy5‐R_8_ or Cy5‐(NlysO)_7_ (10 µM in 1× PBS buffer) for 24 h at room temperature before three rounds of washing with 1× PBS buffer. A slim slice (X: 100‐200 µm, Y: 6 mm, Z: 1000 µm) was cut from the center of each cartilage disk and scanned using a confocal microscope (Zeiss 880). Imaging was acquired at 10× magnification, and only the Z‐stack scans (step depth: 12 µm) from the planes at the very center of the sample were recorded. The zebrafish of 5 days post‐fertilization (dpf) were anesthetized with 0.003% tricaine (Solarbio, T8910) and injected with Cy5‐(NlysO)_7_ (100 µM, 1 nL, 0.1 pmol). All zebrafish were anesthetized 2 h post‐injection for imaging with the EVOS M7000 (Light cubes: Cy5; Objective: 4×) or a confocal microscope. For confocal imaging, zebrafish were fixed in 1% agarose (Macklin, A6338). They were imaged at 10× magnification and Z‐stack scans were obtained with a step depth of 3 µm. Meanwhile, some zebrafish of 5 dpf were embedded in glycerol, stained with Alcian blue (Sigma, 75881‐23‐1) following the manufacturer's instruction, and imaged by a Leica S9D microscope.

### Serum Stability Tests

Cy5‐labeled peptidomimetic probes (10 nmol) were incubated in 25% v/v mouse serum (Biosharp, BL1053A diluted in 1× PBS) at 37 °C. Aliquots (200 µL) were taken periodically at 0 (deadtime: ≈10 min), 2, 24, and 48 h, and added to 400 µL of 3.75% trichloroacetic acid in acetonitrile at 4 °C. Then, all samples were centrifuged to remove the precipitated serum proteins at 13800 g for 10 min at 4 °C. The supernatant (150 µL) was taken to mix with 550 µL of water (with 0.1% TFA) to centrifuge again at 2630 g for 5 min at 4 °C. Five microliter of the supernatant was mixed with 200 µL of water containing 0.1% TFA as the final samples for HPLC analysis at room temperature monitored by absorbance at 646 nm with the integral area of the intact fraction recorded. The peak area for the 10 min time point was designated as 100% intact, and each peak area at successive time points was normalized to the 10 min peak to calculate the percent compound remaining. All the experiments were repeated three times.

### In Vivo and Ex Vivo Fluorescence Imaging in Mice

Typically, the fluorescence peptides or peptoids were prepared at a dose of 1 nmol (10 µM in 100 µL of 1× PBS buffer) and injected into the tail vein of each mouse 2 h prior to fluorescence imaging with an IVIS Spectrum imager (PerkinElmer Lumina III). For Figure 4, Figures S17 and S18 (Supporting Information) (joint aging and RA) and Figure S27 (Supporting Information), 2 nmol of Cy5‐(NlysO)_7_ (20 µM in 100 µL of 1× PBS buffer) were i.v. administered to each mouse. For 1‐week‐old neonatal mice, 1 nmol of Cy5‐(NlysO)_7_ (20 µM in 50 µL of 1× PBS buffer) was intravenously injected into each mouse. All in vivo imaging was performed 2 h post probe injection with the IVIS Spectrum imager (PerkinElmer, fluorescence: ex/em: 620/670 nm, exposure: 1 s, bing: 4, F/Stop: 2; field of view: A/B/C/Z). Mice were anesthesia with 3% isoflurane (RWD, R510‐22‐10) during image acquisition. Hair in the knee areas was removed before fluorescence imaging for mice in Figure 4a and Figure S17 (Supporting Information). The mice were euthanized following the final image acquisition and occasionally imaged again after skin and organ removal. Next, the airways, ankles, ears, paws, knees, tails, spines, and chest ribs were collected, imaged, and saved for subsequent histology analysis. All acquired fluorescence images were analyzed using the Living Image software (PerkinElmer). The fluorescence signal from each specimen was measured by quantifying the average radiation efficiency within the selected oval‐shaped regions‐of‐interest.

For the cartilage retention experiments (Figures S11 and S12, Supporting Information), Cy5‐(NlysO)_7_ (10 µM in 100 µL of 1× PBS buffer) was administered via tail vein to four male Balb/c nu mice (10 weeks old) at 2, 24, 48, and 96 h respectively, prior to fluorescence imaging using an IVIS Spectrum imaging system (ex/em: 620/670 nm, exposure: 1 s, bing: 4, F/Stop: 2). Following euthanasia, their cartilaginous tissues were harvested and imaged. The fluorescence images were analyzed and quantified as described above.

For the biotin delivery experiment (Figure S26, Supporting Information), biotin‐peg3‐K(Cy5)‐Ahx‐(NlysO)_7_ (20 µM in 100 µL of 1× PBS buffer) or 1× PBS buffer (100 µL) was injected into the tail vein of each female Balb/c mouse (20 week‐old) 2 h before fluorescence imaging with an IVIS Spectrum imager (PerkinElmer, fluorescence: ex/em: 620/670 nm, exposure: 1 s, bing: 4, F/Stop: 2). Hair in the knee areas was removed before fluorescence imaging. Next, the mice were sacrificed, and the knee joints were collected and imaged. The femur cartilage was dissected and immersed in Cy3‐streptavidin solution (Jackson ImmunoResearch, 016‐160‐084, 1:200 dilution with 1× PBS buffer) for 2 h at room temperature. After washing with 1× PBS buffer, the femur cartilage was fluorescently imaged with an IVIS Spectrum imager (PerkinElmer, fluorescence: ex/em: 520/570 nm, exposure: 1 s, bing: 4, F/Stop: 2, field of view: A).

### Histology and Microscopy

Paraffin‐embedded tissues were sliced to 5 µm thick using a microtome. Paraffin was removed by xylene, 100% ethanol, 95% ethanol, 85% ethanol, 75% ethanol, 50% ethanol, and PBS buffer for two 5‐min cycles of each solvent. For histological staining, slides of the mouse knee joints were stained with Safranine O / fast green (Solarbio, G1371), Toluidine blue (Servicebio, G1032), or Hematoxylin and Eosin (H&E, Phygene, PH0516) following the manufacturer's instructions. All histology sections were prepared by Servicebio (Wuhan, China). Cryosections of decalcified mouse ankle joints were incubated in Cy5‐(NlysO)_7_ (200 µL, 1 µM in 1× PBS buffer) for 4 h at 4 °C after removing the OCT compound (Figure S18b, Supporting Information). After washing, the sections were sealed in an antifade mounting medium. All fluorescently stained tissue sections were imaged or scanned using an EVOS M7000 imaging system (Light cubes: Cy5, DAPI; Objective: 10×). Nonfluorescent histology slides were scanned with a Pannoramic 250 Flash III scanner (3DHISTECH, 20×).

### Tissue Clearing and Light‐Sheet Fluorescence Microscopy

After injection of 1 or 2 nmol of Cy5‐(NlysO)_7_ and fluorescence imaging at 2 h post‐injection, the mice were perfused with 0.02% m/v heparin (in 1× PBS buffer) and blank PBS solution to remove blood. Next, the samples were collected and cleared following the PEGASOS method.^[^ ^48^ ^]^ After being fixed in 4% paraformaldehyde (PFA) at room temperature for 24 h, the samples were decalcified in 20% EDTA (pH 7.0) solution at room temperature for at least 4 days. The decalcified samples were washed with PBS buffer three times before being decolorized with 25% Quadrol (Sigma, 122262) for 2 days. After being washed three times with PBS buffer, the samples were delipidated with tert‐butanol (Sigma, 360538) and dehydrated with 70% v/v tert‐butanol, 27% v/v PEGMEMA500 (Sigma, 447943), and 3% w/v Quadrol for 2 days. Finally, the samples were immersed in a clearing medium (BB‐PEG) made by 75% v/v benzyl benzoate (Sigma, W213802), 22% v/v PEGMEMA500, and 3% w/v Quadrol for at least 2 days. All steps were performed in the dark.

Each cleared specimen was imaged on a LaVision Biotec Ultramicroscope Blaze equipped with an sCMOS camera (ex/em: 630/680 nm). The tissue was immersed in the imaging chamber filled with the BB‐PEG medium. Each cleared specimen was scanned with a magnification of 4 on both sides, each side composed of three light‐sheet beams with a 5 µm step in the Z‐axis. The images were acquired by continuous light‐sheet scanning, and stitched using a blend algorithm on both sides. Images were acquired by ImSpector (LaVision BioTec), saved as 16‐bit grayscale TIFF images for each channel, and reconstructed with the Imaris software. Movies were produced at a frame rate of 25 fps.

### Biosafety Tests—Cytotoxicity

The cultures were maintained under standard conditions of 37 °C and 5% CO_2_. Chondrocytes collected from the articular cartilage of Sprague‐Dawley (SD) rats (purchased from Jennio Biotech, JNO‐720) were cultured in DMEM medium (Precell, PM150210). Human venous endothelial cells (HUVECs, purchased from iCell, iCell‐h110) were cultured in HUVEC medium (iCell, iCell‐h110‐001b). Both cells were seeded in 96‐well plates at a density of 5000 cells per well. The medium was supplemented with 10% fetal bovine serum (FBS, Precell, 164210) and penicillin‐streptomycin (1:100 diluted from a 100× stock solution, ThermoFisher, 15140122) to support cell growth and prevent bacterial contamination. When cell confluency reached 80%, peptidomimetic probes at increasing concentrations ranging from 0 to 40 µM were added to the cell culture medium (*n* = 4 wells for chondrocytes, *n* = 6 wells for HUVECs; blank control group: 0 µM; cells cultured without probe). Following 24 h (or 72 h) of incubation, the medium was removed, and the cells were rinsed three times with 1× PBS buffer. Cell viability was assessed using the CCK‐8 assay kit (GLPBIO, GK10001−5) according to the manufacturer's instructions. After further incubation for 4 h, the optical density (OD) of the CCK‐8 solution was measured at 450 nm using a microplate reader (PerkinElmer, EnVision). For live‐dead cell staining, rat chondrocytes (5000 cells per well seeded, *n* = 4 wells per group) were cultured for 24 h in medium (without fetal bovine serum) containing 40 µM of each peptidomimetic probe in a 96‐well plate. Subsequently, the medium was removed, and the cells were stained using a Calcein/PI cytotoxicity assay kit (Beyotime, C2015M) directly without washing, following the manufacturer's instructions. The fluorescence images were obtained using an EVOS M7000 imaging system (Light cubes: GFP and RFP, Objective: 10×). A microscopic image was taken from the live (Calcein AM) staining of each well, and the total fluorescence signals were quantified for each image. The mean fluorescence level of each probe's group was normalized to the blank control group to estimate the relative cell viability. For cellular uptake tests, 10 µM of each compound was added to the cell culture medium (*n* = 3 or 5 wells; blank control group: 0 µM, cells cultured without probes). Following 2 h or 24 h of incubation, the medium was removed, and the cells were rinsed three times with 1× PBS buffer. The fluorescence intensity of each well was measured by the microplate reader (Ex / Em: 620 /662 nm). Rat chondrocytes were further incubated with Hoechst 33342 (Thermo Fisher, R37605, 2 drops per milliliter) for 20 min at room temperature and imaged using an EVOS M7000 imaging system (Light cubes: Cy5, Objective: 20×). Additionally, the chondrocytes were fixed with 4% PFA for 15 min and incubated with 0.1% Triton X‐100 for 5 min. Phalloidin‐iFluor 555 (Abcam, ab176756; 1:500 dilution with 1× PBS buffer) was then added to the wells, and the cells were incubated for 1 h at room temperature. After washing with 1× PBS buffer three times, cells were imaged using a confocal microscope to visualized the cellular uptake of the Cy5‐labeled probes [Zeiss 880; Ex / Em (hoechst): 405 / 470 nm; Ex / Em (phallodin): 543 / 584 nm; Ex / Em (Cy5‐labeled probes): 633 / 699 nm; Objective: 40×].

### In Vivo Biocompatibility Tests

Whole blood tests: Each C57BL/6J mouse (6–8 weeks old) was intravenously injected with 100 µL of PBS buffer or with each peptide or peptoid probe (2 nmol in 100 µL 1× PBS buffer per mouse) on days 0, 7, and 14. The mice were sacrificed on day 15, 24 h after the final injection. The heart, liver, spleen, lungs, and kidneys were collected from each mouse and processed into formalin‐fixed paraffin‐embedded sections for histological evaluation with H&E staining. Whole blood was collected. Both H&E and blood sample analyses were performed by Wuhan Servicebio.

Blood biochemical analysis: Each C57BL/6J mouse (6–8 weeks old) was intravenously injected with 100 µL of PBS or Cy5‐(NlysO)_7_ (2 nmol in 100 µL 1× PBS buffer per mouse) 24 h prior to blood collection for the biochemical analysis. Biochemical analysis of the blood was performed by Wuhan Servicebio.

### Data Analysis and Statistics

GraphPad (version 9.0.0) was used to analyze and plot the experimental data. As shown in figure legends, numbers were represented as median with interquartile range (for non‐normally distributed data), along with the mean with standard error of the mean (s.e.m.) (for normally distributed data). A paired t‐test was used to compare two groups of paired normally distributed data. One‐way analysis of variance (ANOVA) with a *post hoc* Tukey HSD test was used for data indicating homoscedasticity, and Welch's ANOVA with post‐hoc Dunnett's T3 test was used for data indicating heteroscedasticity. For multiple comparisons among group means of abnormally distributed data, the Kruskal‐Wallis test with Dunn's multiple comparisons test was used. *P* values below 0.05 were regarded as statistically significant and *P* values above 0.05 were not shown in any figures unless noted.

## Conflict of Interest

The authors declare no conflict of interest.

## Author Contributions

C.Z. and R.Q. contributed equally to this work. Y.L. performed conceptualization. C.Z., S.Z., R.Q., K.H., Y.H., Y.H.L. (Yinghua Liu), and Y.L. performed methodology. C.Z. and R.Q. performed investigation. C.Z., S.Z., R.Q., and Y.L. performed data acquisition/analysis. C.Z., S.Z., R.Q., and Y.L. performed visualization. Y.L. performed resources. Y.L. and S.Z. performed supervision. Y.L., C.Z., S.Z., and R.Q. wrote—original draft. C.Z., S.Z., R.Q., and Y.L. wrote—reviewed and edited.

## Supporting information



Supporting Information

Supplemental Video 1

Supplemental Video 2

## Data Availability

The data that support the findings of this study are available from the corresponding author upon reasonable request.
