# Supplementary material for: Cationic Peptoids for Systemic In Vivo Cartilage‐Targeting
Source: Adv Sci (Weinh). 2025 Aug 31;12(43):e02781. doi: 10.1002/advs.202502781 (PMC12631873; doi:10.1002/advs.202502781)
Supplement: Supplementary file 1 — Supporting Information [file ADVS-12-e02781-s001.pdf]

Supporting Information

Cationic peptoids for systemic in vivo cartilage-targeting

Authors

Chaonan Zhang[1,#], Rongmao Qiu[1,2,#], Yongjie Huang[1], Yinghua Liu[1], Kui Huang[1], Suwen Zhao[1,], Yang Li[1,*]*

Affiliations

[1]Guangdong Provincial Engineering Research Center of Molecular Imaging, Guangdong-Hong Kong-Macao University Joint Laboratory of Interventional Medicine, the Fifth Affiliated Hospital, Sun Yat-sen University, Zhuhai, Guangdong 519000, China.

[2]Current address: School of Pharmacy and Medical Technology, Key Laboratory of Pharmaceutical Analysis and Laboratory Medicine of Fujian Province, Putian University, Putian, Fujian 351100, China.

*Corresponding authors.

Emails: liyang266@mail.sysu.edu.cn (Yang Li); zhaosw5@mail.sysu.edu.cn (Suwen Zhao)

[#]These authors contributed equally to this work.

Contents

Supplementary Methods

Supplementary Figures S1-S27

Supplementary Videos 1-2

Supplementary Methods

Solid phase synthesis and purification

All Materials and reagents were purchased from commercial sources and used without further purification. All sequences were prepared on Rink Amide AM resin (HECHENG, loading: 0.37 mmol/g) on a PurePep Chorus peptide synthesizer. During the swelling, reaction, or wash steps, nitrogen gas was passed into the reactor to agitate the resin slurry. The amino acid building blocks, including Fmoc-Arg(pbf)-OH (Macklin, F809667), Fmoc-Hyp(tBu)-OH (Aladdin, F117058), Fmoc-Gly-OH (Aladdin, F103019), Fmoc-Lys(Boc)-OH (GL Biochem, 36802), Fmoc-Ahx-OH (Aladdin, F117709), were coupled following standard Fmoc procedures. Typically, the resin (135 mg, 0.05 mmol) was swollen in 3 mL DMF (Aladdin, D112002) for 10 min. Fmoc-deprotection was performed with 3 mL 20% (v/v) piperidine (Detian fine chemicals, 20191105) in DMF for 90 s at 50 °C. Following each reaction, the resin was drained and washed with 3 mL DMF three times. Coupling of all amino acids was performed by agitating the resin with a solution of 0.25 mmol Fmoc amino acid, 0.25 mmol HATU (Aladdin, H109327), 0.25 mmol HOAT (Macklin, H811122), and 0.50 mmol DIEA (EMD Millipore, S1807494945) in 4 mL of DMF for 5 min at 50 °C twice (resin: amino acid: HATU: HOAT: DIEA = 1: 5: 5: 5: 10).

Peptoid residue Nlys was incorporated on-resin by the submonomer method: (1) acylation was performed by the addition of a solution of 0.49 mmol N,N'-diisopropylcarbodiimide (DIC, Aladdin, D106162) and 0.50 mmol bromoacetic acid (Macklin, B802565) in 2 mL DMF to 0.05 mmol of resin-bound amine for 30 min twice (resin: DIC: bromoacetic acid = 1: 9.8: 10); (2) after washing the resin with 3 mL DMF three times, 1.00 mmol N-Boc-1,4-diaminobutane (Macklin, N802045) in 2 mL DMF was added and the reaction mixture was stirred for 60 min at 50 °C.

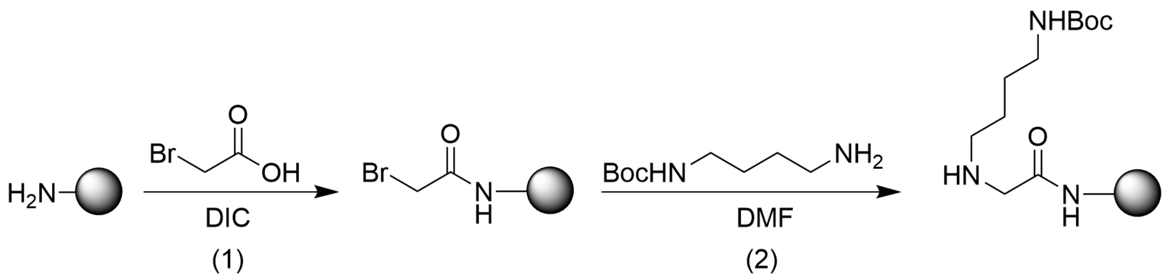

Following each reaction, the resin was drained and washed with 3 mL DMF three times. If the next residue was Gly, Hyp, or Ahx, the HATU/Fmoc coupling method was used.

Fluorescence labeling of the sequences were achieved by treating the peptide/peptoid-bound resin (3.20 μmol) with sulfo-Cyanine 5 succinimidyl ester (1.28 μmol, designed as Cy5-NHS, Lumiprobe, 63220) and DIEA (6.40 μmol) in 90 μL dimethyl sulfone (DMSO, Aladdin,

D106264) for over 24 h at room temperature in the dark. The final cleavage was carried out by treating the resin with a TFA (Macklin, T818782-500ml)/TIS/water (95:2.5:2.5) mixture for 3 h (6 h for Cy5-R_8 to allow sufficient removal of the sidechain protective group pbf). The TFA cleavage solution was collected and evaporated under a stream of nitrogen down to approximately 0.5 mL. Crude products were precipitated by adding excess cold ethyl ether to the TFA solution, followed by centrifugation (4000 rpm for 4 min) at 4 °C.

The crude peptides or peptoids were purified by reverse-phase high-performance liquid chromatography (RP-HPLC) on a semi-preparative column (Agilent ZORBAX StableBond 300 C18) using a linear gradient of 5% to 50% acetonitrile in 25 min at room temperature. The purified fractions were collected, lyophilized, and reconstituted in Milli-Q water as stock solutions. All purified peptides or peptoids were verified by matrix-assisted laser desorption ionization-time of flight mass spectrometry (MALDI-TOF MS, Shimadzu 8020). The concentrations of stock solutions were determined using absorbance at 646 nm (extinction coefficient: 271000 M^{-1} cm^{-1}) on an Implen NP80 spectrophotometer. The ^{1}H NMR spectra of peptidomimetics were measured in DMSO-d_6 on a Bruker AVANCE 500 MHz at 25 °C.

Supplementary Figures

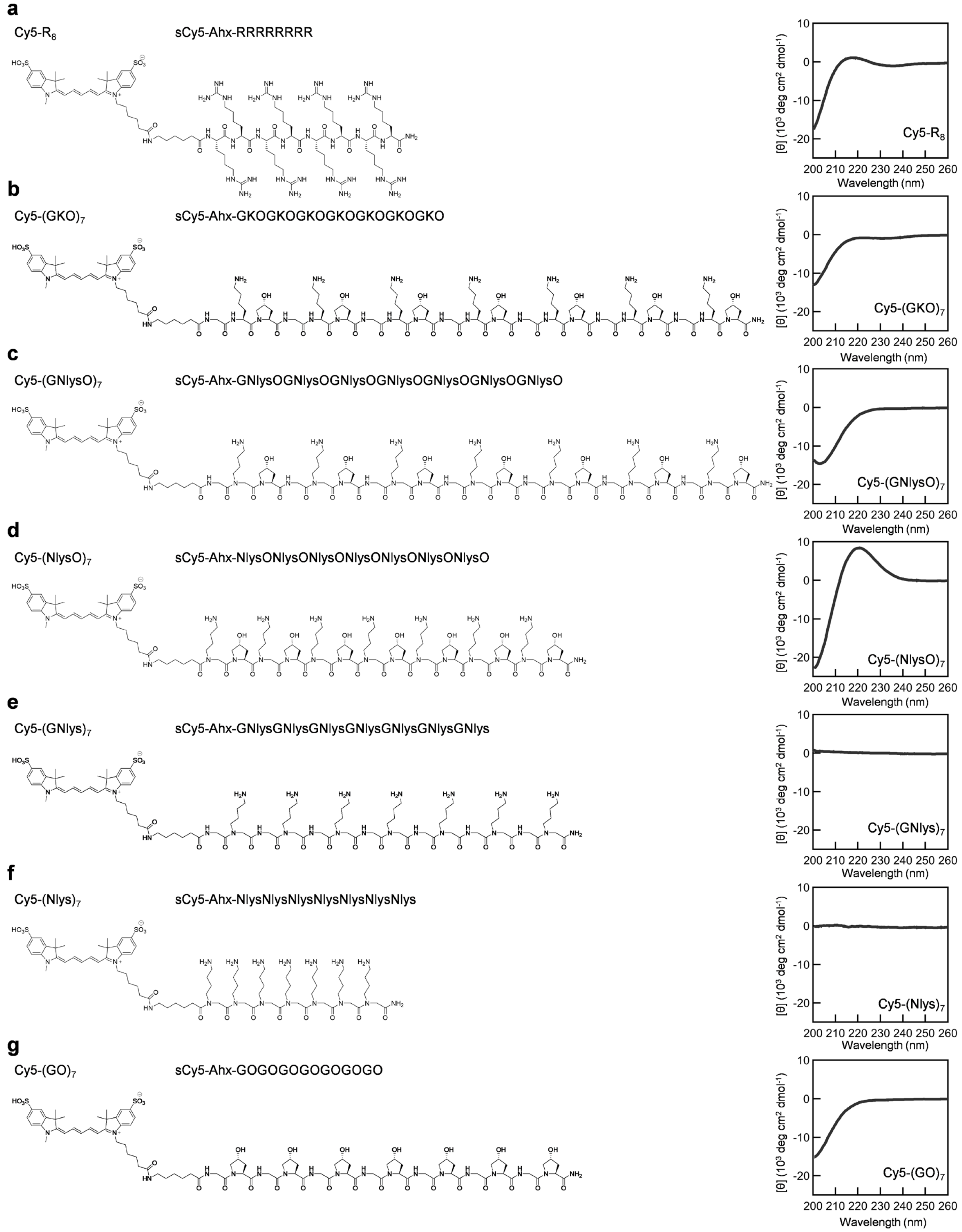

Figure S1. Chemical structures and circular dichroism (CD) spectra of all peptidomimetics (measured at 25 °C in 1×PBS buffer, pH 7.4). The CD spectrum of Cy5-(NlysO)$_7$ displays a characteristic positive band at 220–230 nm suggesting a poly-proline II helix conformation.

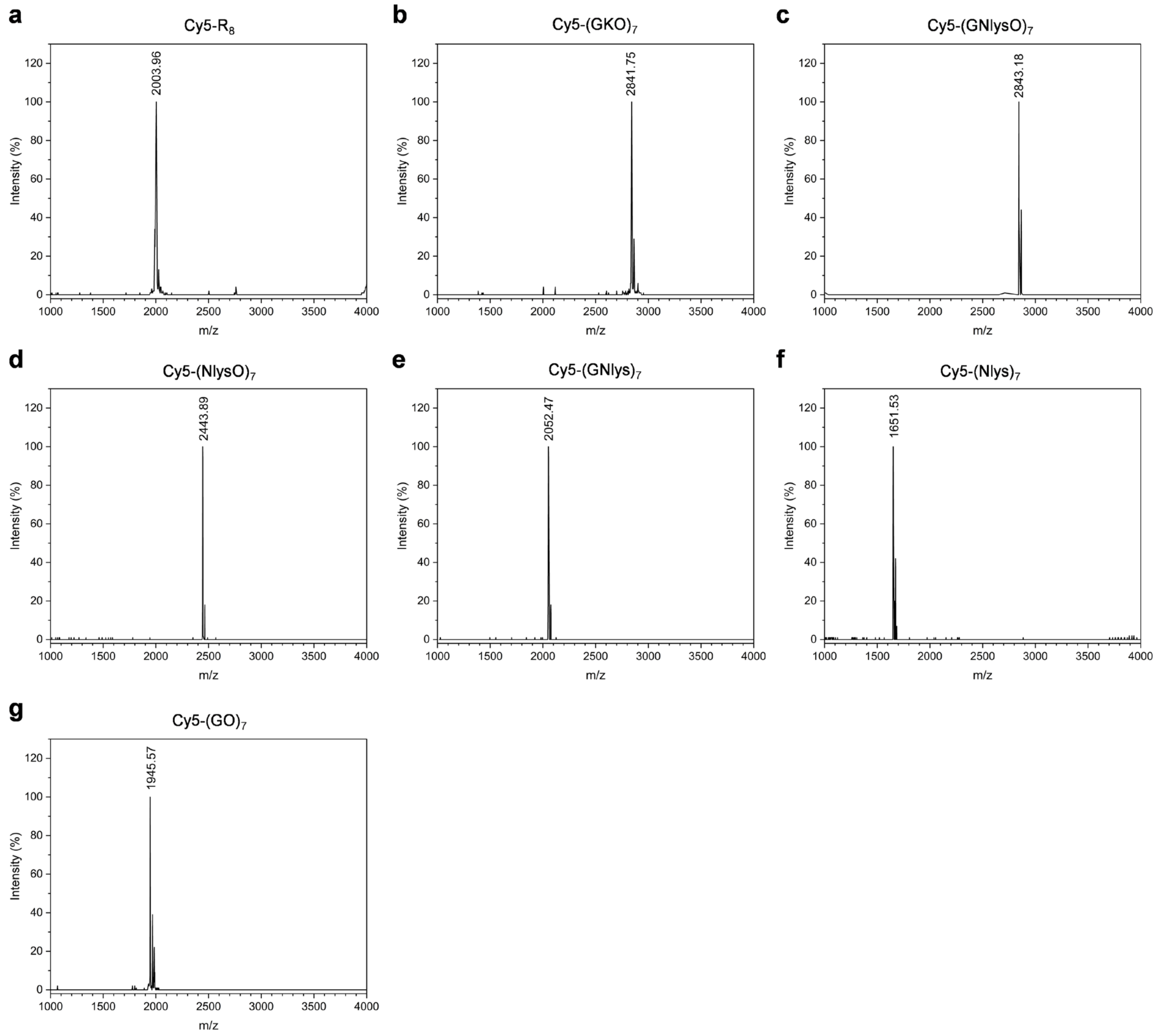

Figure S2. The MALDI-MS spectra of all peptidomimetics. **a.** Cy5-R_8, calculated: 2004.12 $[M+H]^+$, observed: 2003.96 $[M+H]^+$. **b.** Cy5-$(GKO)_7$, calculated: 2842.46 $[M+H]^+$, observed: 2841.75 $[M+H]^+$. **c.** Cy5-$(GNlysO)_7$, calculated: 2842.46 $[M+H]^+$, observed: 2843.18 $[M+H]^+$. **d.** Cy5-$(NlysO)_7$, calculated: 2443.31 $[M+H]^+$, observed: 2443.89 $[M+H]^+$. **e.** Cy5-$(GNlys)_7$, calculated: 2051.13 $[M+H]^+$, observed: 2052.47 $[M+H]^+$. **f.** Cy5-$(Nlys)_7$, calculated: 1651.97 $[M+H]^+$, observed: 1651.53 $[M+H]^+$. **g.** Cy5-$(GO)_7$, calculated: 1945.79 $[M+H]^+$, observed: 1945.57 $[M+H]^+$.

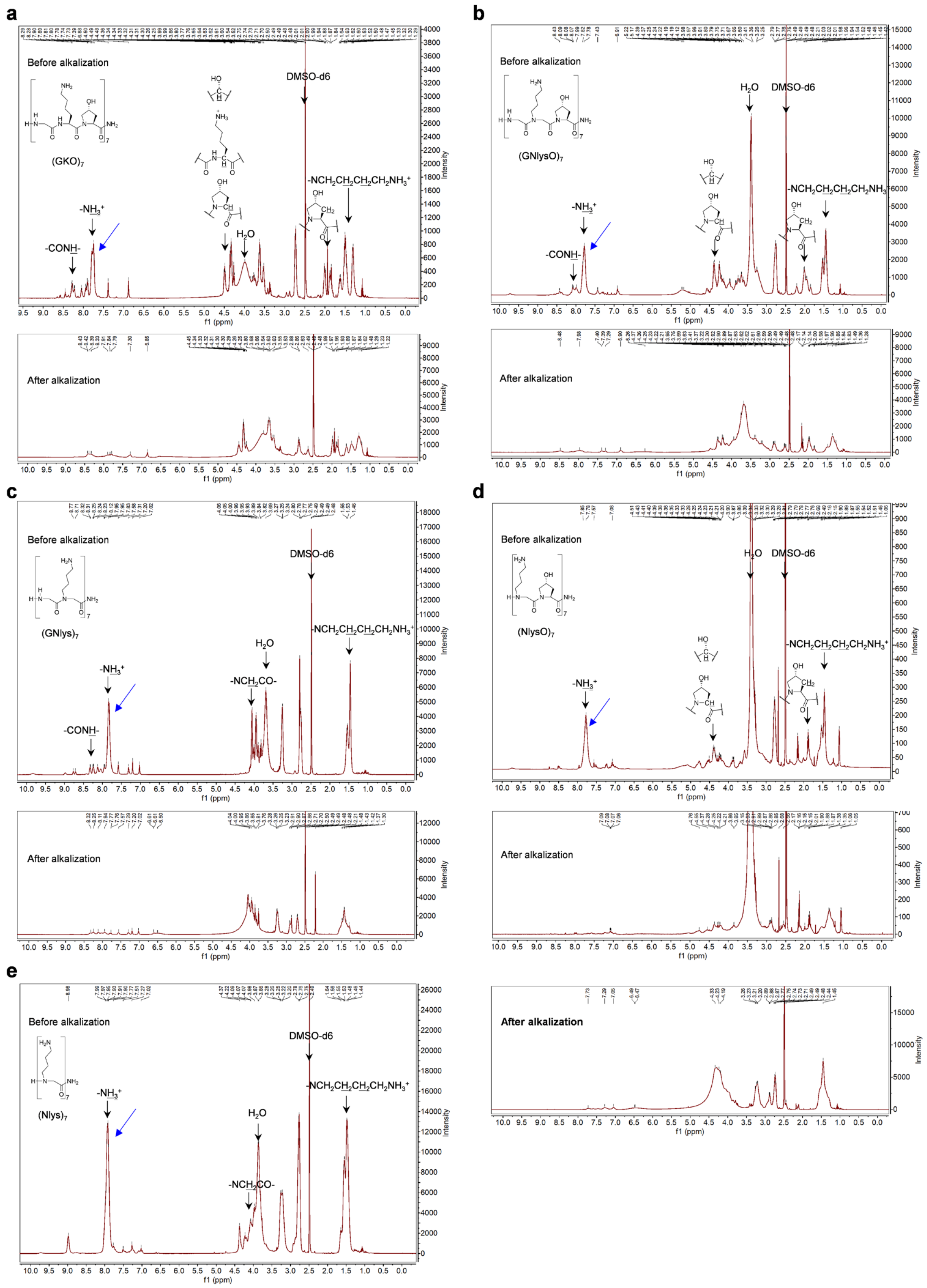

Figure S3. The ^{1}H NMR spectra of $(GKO)_7$ and the Nlys-containing sequences in DMSO-d_6 before (top) and after (bottom) alkalization. After adjusting the pH of the solutions from acidic to above 10 with sodium carbonate, the notable peaks near 7.8 ppm representing the hydrogen atoms in -NH_3^+ (blue arrows) all disappeared. Key peaks: amides from Gly or Lys (8.0-8.5 ppm) (e.g., a,b,c); methynes from Lys or Hyp residues (4.0-4.5 ppm) (e.g., a,b,d)**;** methylenes from Gly or Nlys's α-carbons (~4.0 ppm) (e.g., c,e); methylenes from Lys or Nlys's amino sidechains (1.5 ppm) (e.g., a-e).

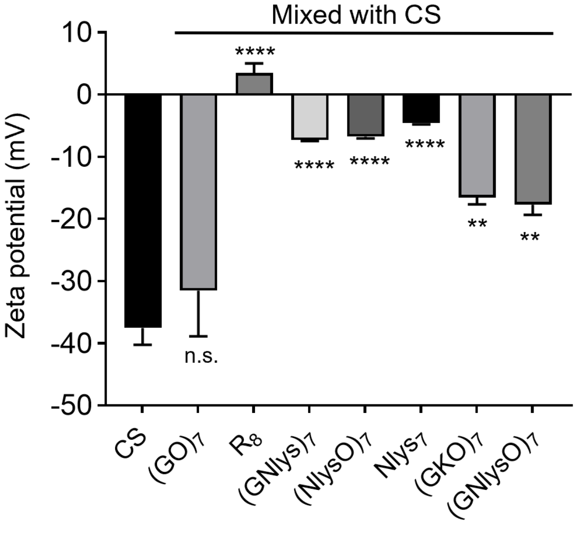

Figure S4. Zeta potential measurements of chondroitin sulfate sodium (CS, 0.02 mg/mL) in pure water (1 mL) with or without adding the peptidomimetic compounds (11.6 nmol, n = 3 samples) at room temperature. The negative value of the zeta potential of the CS solution significantly decreased only after being mixed with each cationic probe. Data (mean + s.e.m.) were compared with the CS only group using a one-way ANOVA with *post hoc* Tukey HSD test (**: $P < 0.01$, ****: $P < 0.0001$, n.s. = not significant: $P > 0.05$).

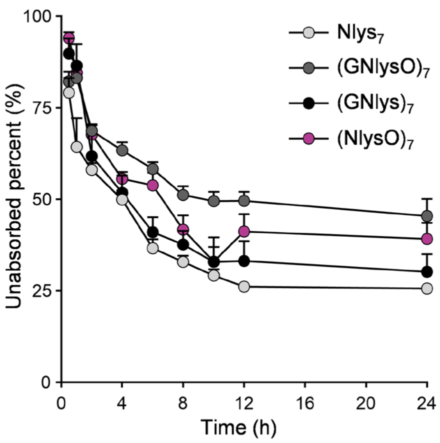

Figure S5. In vitro cartilage absorption curves of Cy5-(GNlys)$_7$, Cy5-Nlys$_7$, Cy5-(GNlysO)$_7$, or Cy5-(NlysO)$_7$. Porcine cartilage explant plugs (10 mg each, n = 6 individual samples) were incubated in PBS solutions of the four peptidomimetics (10 µM, 200 µL) over 24 h at room temperature. The unabsorbed percentages of the probes were calculated from the measured fluorescence signals left in the solutions at designated time points. The cartilage absorption of these four peptoids appeared nearly saturating after 12 to 24 h. Data: mean + s.e.m.

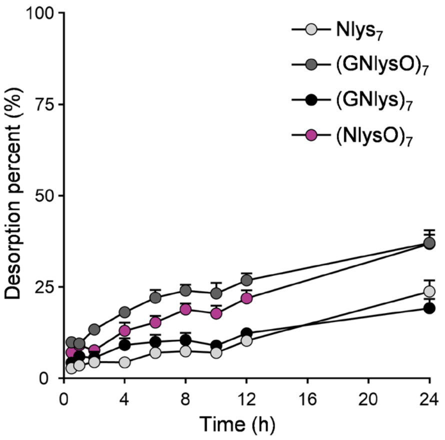

Figure S6. In vitro desorption curves of Cy5-(GNlys)$_7$, Cy5-Nlys$_7$, Cy5-(GNlysO)$_7$, and Cy5-(NlysO)$_7$ from the porcine cartilage explant plugs over 24 h in 1× PBS buffer at room temperature following 24 h of absorption. The desorption percentages were calculated by the measured Cy5 fluorescence signals from the washing buffer. Only approximately 20-40% of these peptoid probes were released within 24 h. Data: mean + s.e.m. (*n* = 6 individual samples).

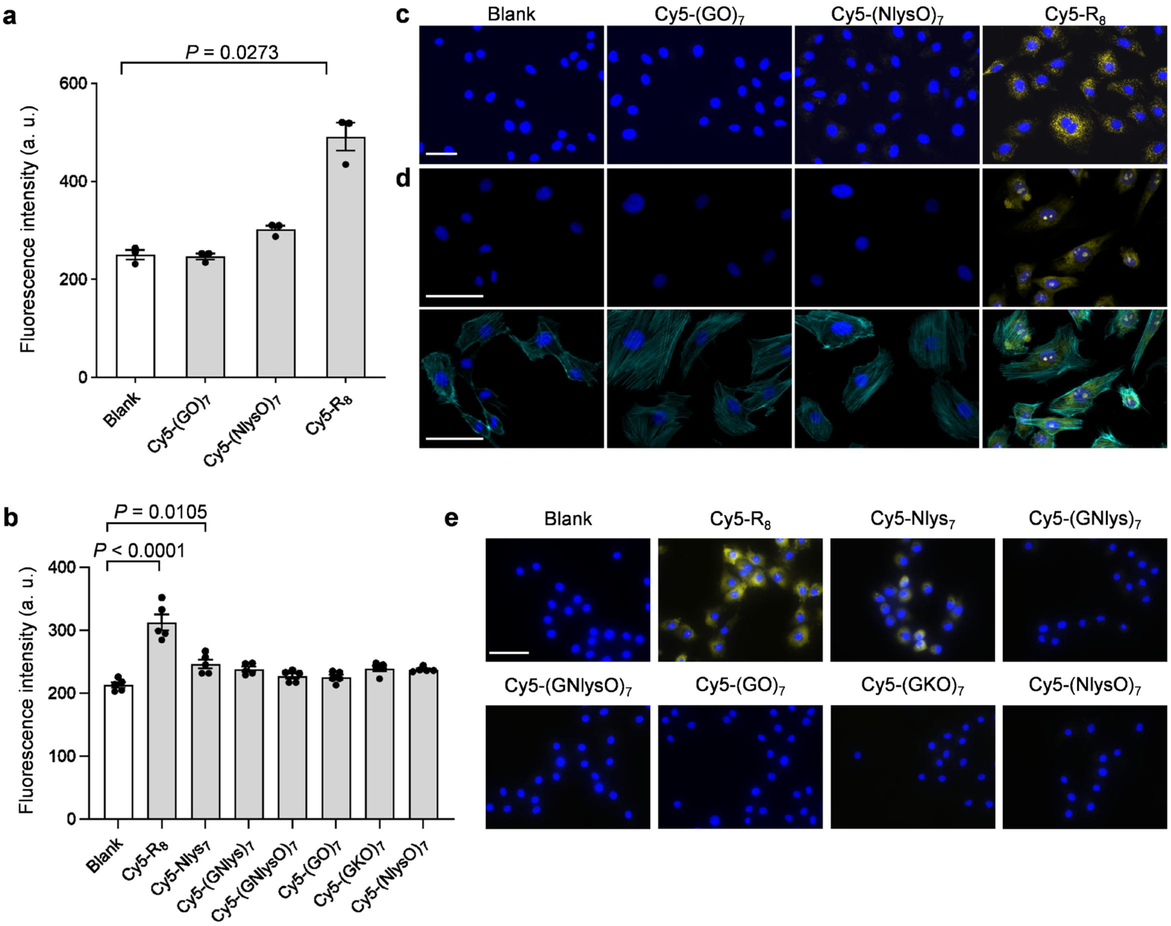

Figure S7. The cellular uptake of Cy5-R_8 by the chondrocytes. **a,b.** Fluorescence intensity of a 96-well plate in which rat chondrocytes were cultured. The fluorescence intensities were measured by a microplate reader after the cells were rinsed following 2 h (a) or 24 h (b) incubation with no probe (blank), or with 10 µM of the peptidomimetics, or Cy5-R_8 in the culture medium. Data are displayed as mean ± s.e.m. (data points: individual wells) and analyzed using one-way ANOVA with *post hoc* Tukey HSD test. **c-e.** Representative fluorescence micrographs of live rat chondrocytes co-stained with Hochest 33342 (c, e) and fixed chondrocytes co-stained with Hochest 33342 and Phaodin-iFluor 555 (d) following 2 h (c, d) or 24 h (e) incubation with no probe (blank), or with 10 µM of the peptidomimetics, or Cy5-R_8 in the culture medium. These data and images from the 24 h time point (b, e) showed the strong cellular uptake of Cy5-R_8 and some weak uptake of Cy5-$Nlys_7$ (e) compared to the negligible uptake of Cy5-$(GNlys)_7$, Cy5-$(GNlysO)_7$, Cy5-$(GO)_7$, Cy5-$(GKO)_7$, and Cy5-$(NlysO)_7$ by the chondrocytes. Blue: cell nucleus (Hoechst 33342); Green: cytoskeleton (Phaodin-iFluor 555); Yellow: Cy5-labeled probes. Scale bars: 50 µm (c, d) and 75 µm (e).

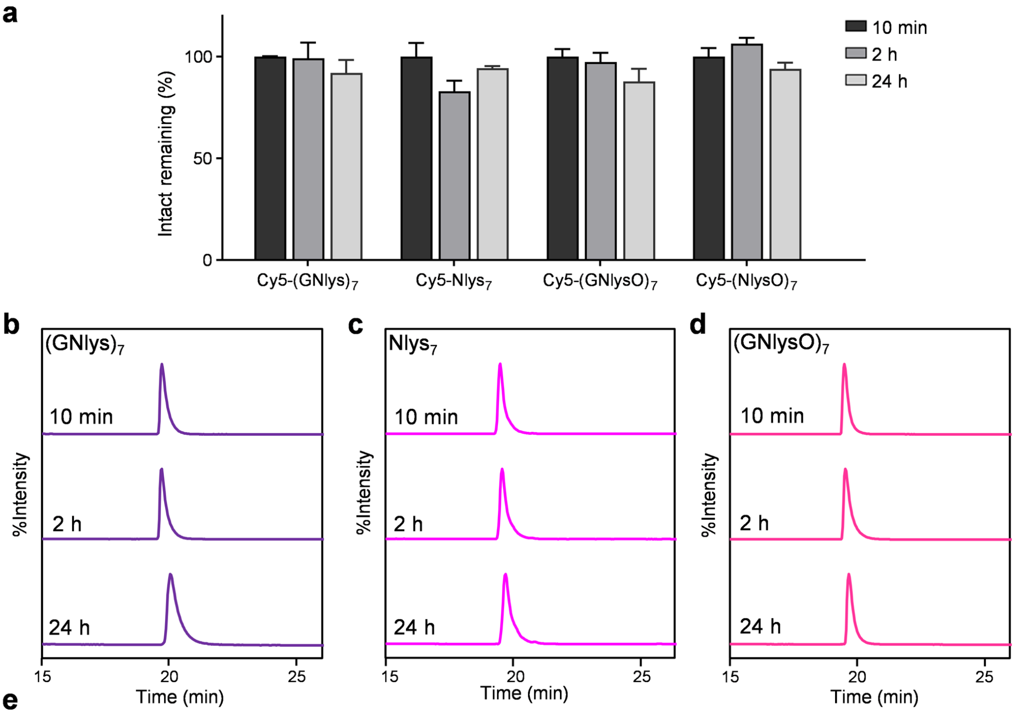

e

Sequence	MS (Exact mass)	Fragment HPLC peak*	Elution time (min)	Measured MALDI-MS
Cy5-R_8	2003.12	pk1	20.43	912.57
	2003.12	pk2	21.30	757.81
Cy5-(GKO)$_7$	2841.46	pk1	19.93	1111.66
	2841.46	pk2	20.54	813.48
Cy5-(GO)$_7$	1944.79	pk1	20.18	926.65

Figure S8. Additional serum stability data of the peptoid sequences. **a.** Percent of the intact compound for Cy5-(GNlys)$_7$, Cy5-Nlys$_7$, Cy5-(GNlysO)$_7$, and Cy5-(NlysO)$_7$ measured by HPLC after incubation in mouse serum at 37 °C for 10 min, 2 h, and 24 h (n = 3 samples, data: mean + s.e.m.) **b-d.** The representative HPLC chromatograms (monitored with optical absorbance at 646 nm) of Cy5-(GNlys)$_7$, Cy5-Nlys$_7$, Cy5-(GNlysO)$_7$ at different time points during serum incubation. No significant changes in peak shape and elusion time were noted post 24 h of serum incubation. *: sequence-fragment peak. **e.** The calculated MS (exact mass) of the full-length sequences and the measured MALDI-MS of the sequence fragments eluted from the unexpected HPLC peaks appeared in the serum stability HPLC runs for Cy5-R_8, Cy5-(GKO)$_7$, and Cy5-(GO)$_7$. The peak numbers for each compound in this table match with the ones in Figure 3a and Figure S4c.

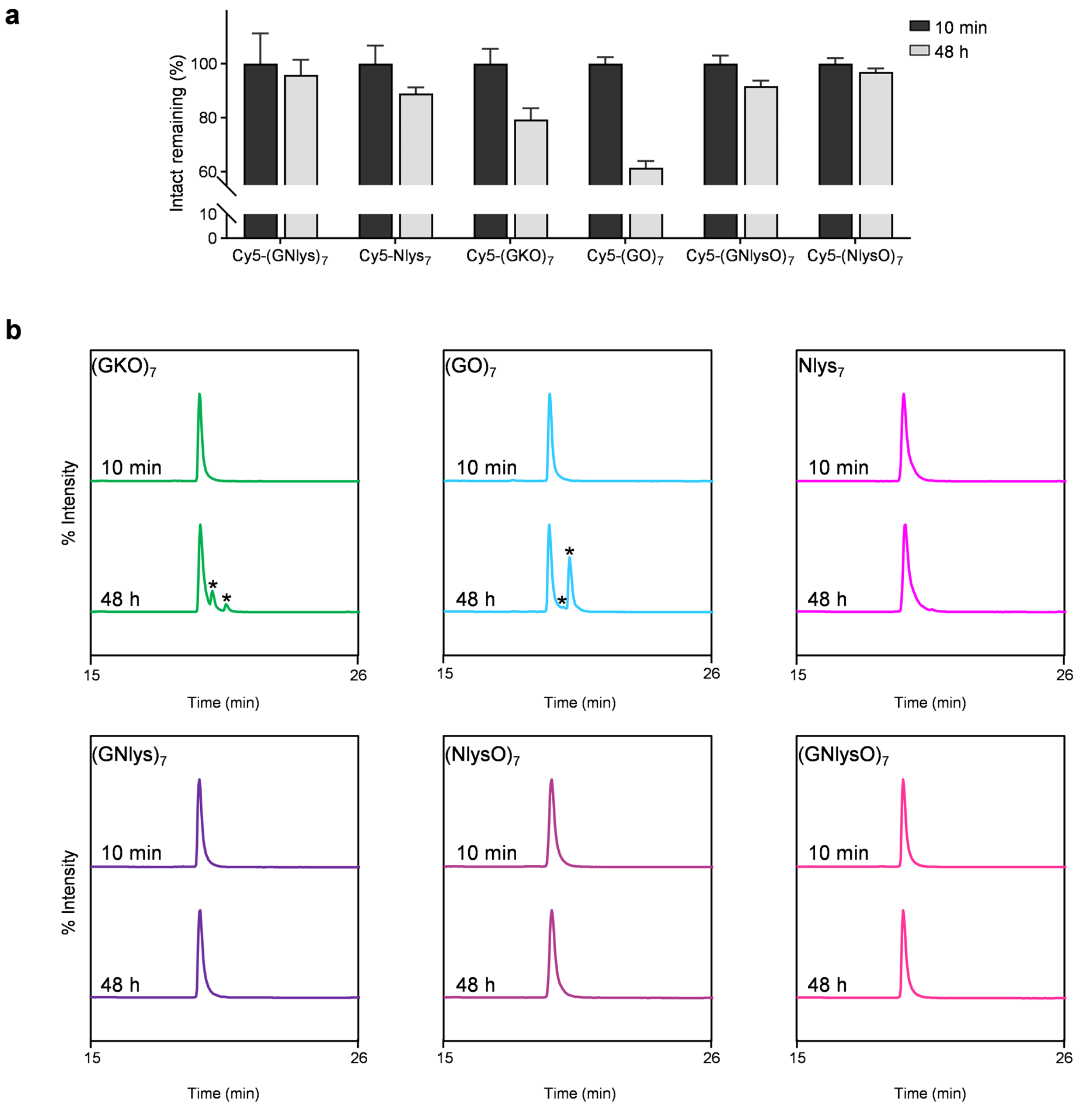

Figure S9. Serum stability of the peptidomimetics after 48 h incubation. **a.** Percent of the intact compound for Cy5-(GNlys)$_7$, Cy5-Nlys$_7$, Cy5-(GKO)$_7$, Cy5-(GO)$_7$, Cy5-(GNlysO)$_7$, and Cy5-(NlysO)$_7$ measured by HPLC after incubation in 25% mouse serum at 37 °C for 10 min or 48 h (n = 3 samples, data: mean + s.e.m.) **b.** The representative HPLC chromatograms (monitored with optical absorbance at 646 nm) of the six compounds after 10 min or 48 h 25% serum incubation. No significant changes in peak shape and elution time were noted for the Nlys-rich peptidomimetics even post 48 h of serum incubation, while Cy5-(GO)$_7$ and Cy5-(GKO)$_7$ showed prominent peaks from peptide proteolysis (*: fragment peak).

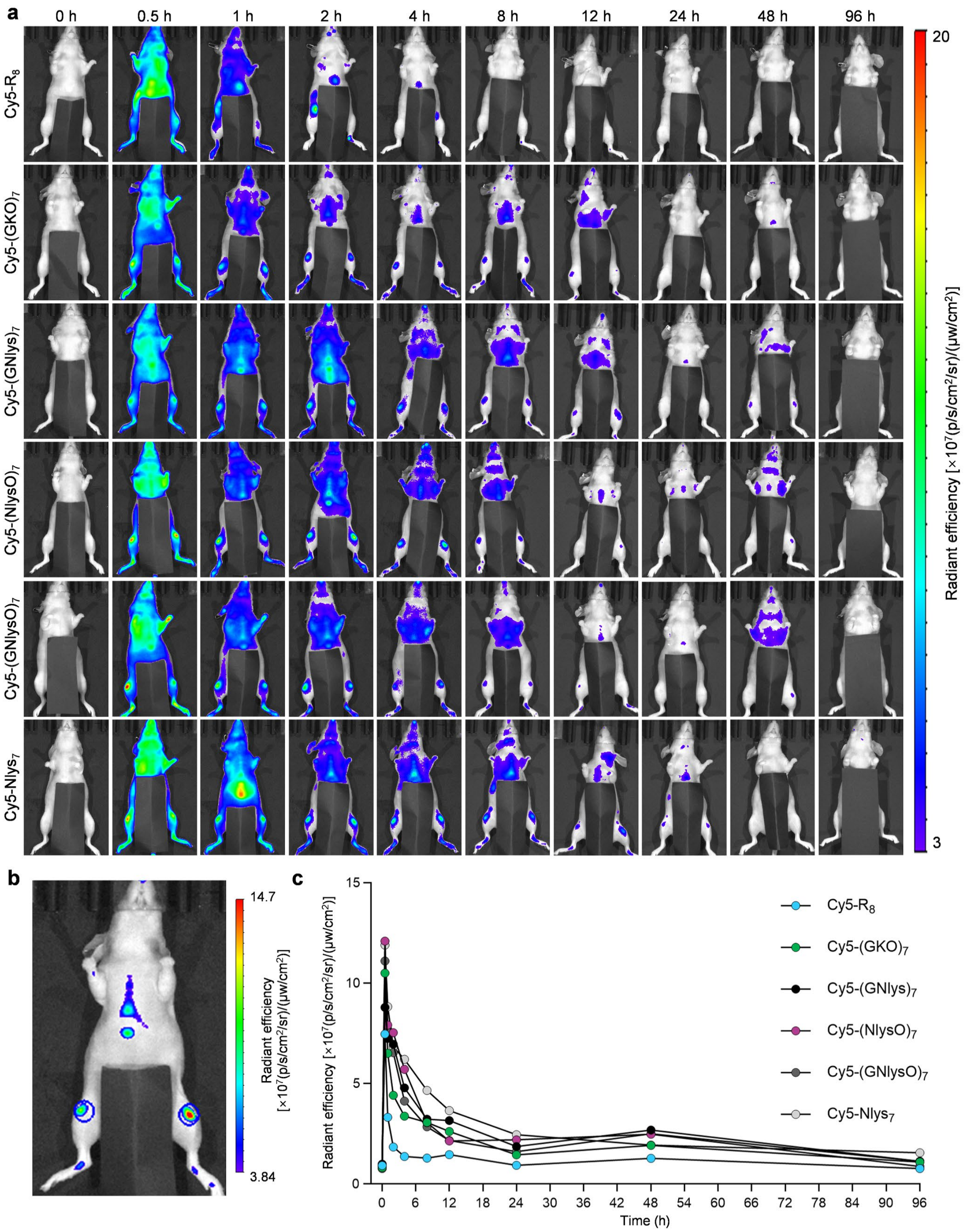

Figure S10. a. The in vivo fluorescence images of nude mice (8 weeks old) intravenously injected with 1 nmol of each peptidomimetic probe at various time points pre- (0 h) and post-injection (until 96 h). Nlys-containing sequences Cy5-(NlysO)$_7$, Cy5-(GNlysO)$_7$, Cy5-(GKO)$_7$, Cy5-(GNlys)$_7$, and Cy5-Nlys$_7$ showed stronger retention in the joints and ribs in vivo compared to the peptide sequences Cy5-(GKO)$_7$ and Cy5-R$_8$, as well as clearance of most of the fluorescence signals from the cartilage in 48 h post-injection. **b.** Regions-of-interest at the knee joints were selected for fluorescence quantification. **c.** Quantified mean fluorescence signals in the regions-of-interest at the knee joints of the mice in a. Each point in c represents the average of the quantified signals of the two knees of each mouse.

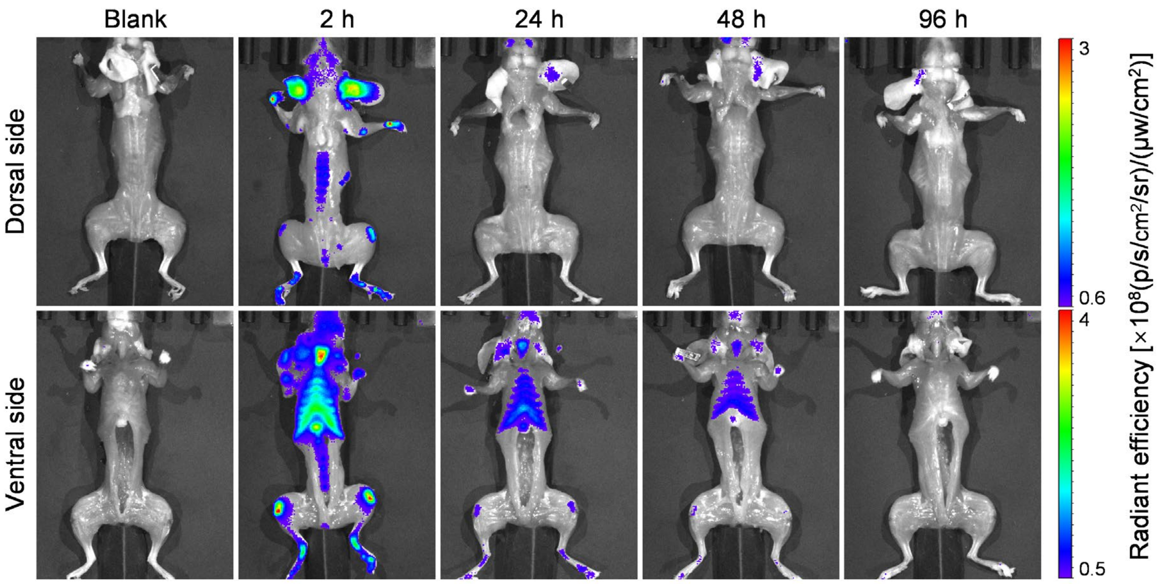

Figure S11. In vivo retention of Cy5-(NlysO)$_7$ in the mouse cartilage. The fluorescence images of the musculoskeletal tissues of each nude mouse were acquired at 2, 24, 48, and 96 h post intravenous injection of 1 nmol of Cy5-(NlysO)$_7$. Weak fluorescence signals could still be observed in the cartilage-rich tissues, including the knee joints and ribs, at 48 h but not 96 h post-injection.

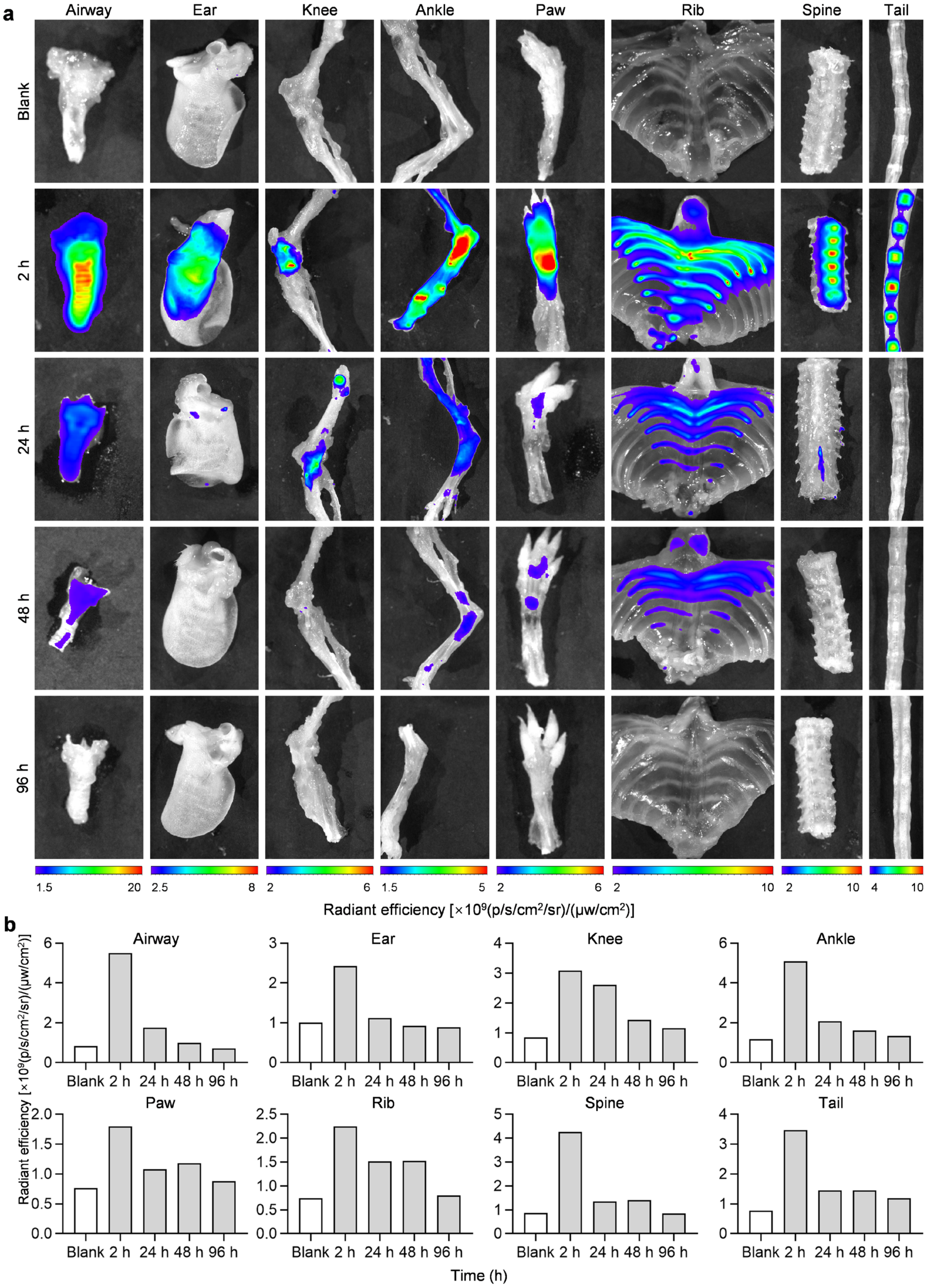

Figure S12. a. Representative fluorescence images of the cartilaginous tissues collected from the mice in Figure S11. **b.** Quantified fluorescence signals in the same regions-of-interest of each of the cartilaginous tissues harvested at different time points in a.

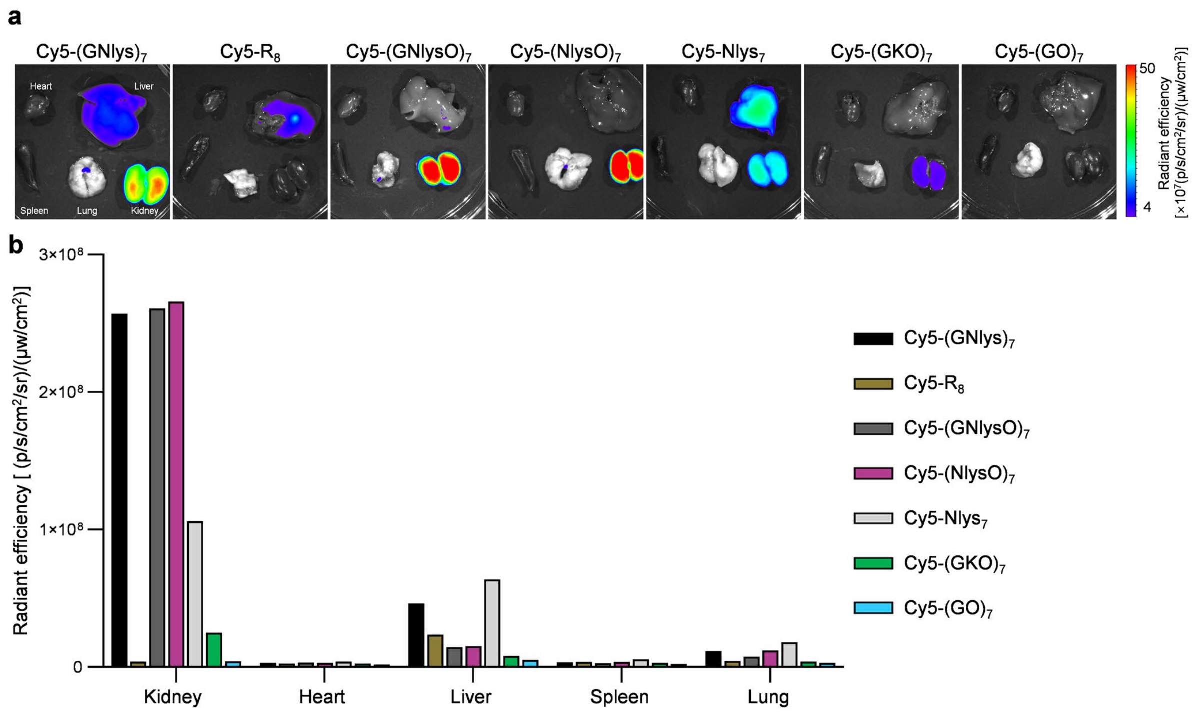

Figure S13. a. Fluorescence images of the organs isolated from the nude mice 2 h post intravenous injection of 1 nmol of each peptidomimetic probe. **b.** Quantitative analysis of each probe's fluorescence signals in the organs displayed in **a**. From these images and data, Cy5-(NlysO)$_7$, Cy5-(GNlysO)$_7$, and Cy5-(GKO)$_7$ might be mainly cleared through kidneys; Cy5-(GNlys)$_7$ and Cy5-Nlys$_7$ may be cleared by the kidneys and liver.

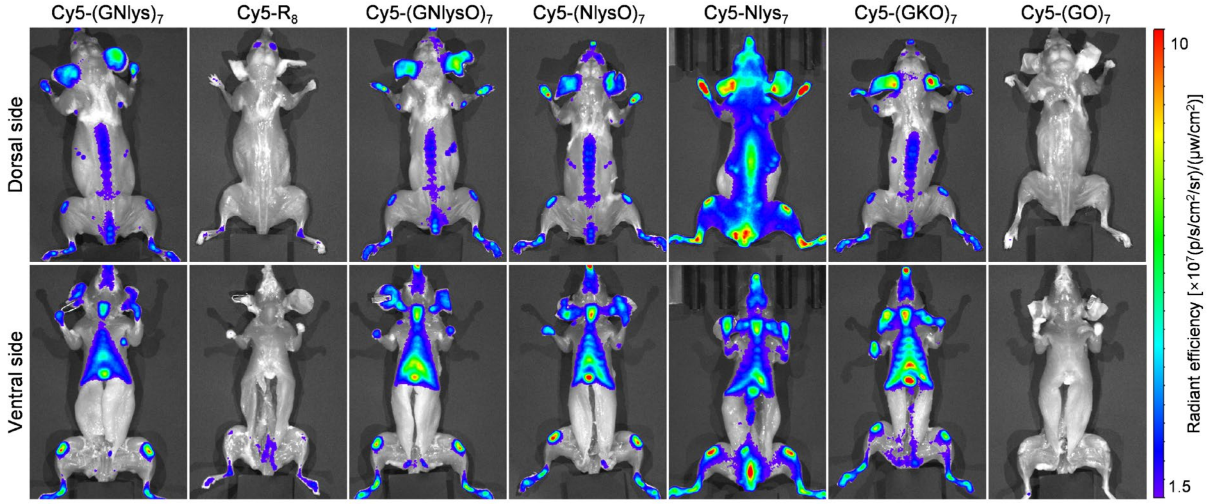

Figure S14. In vivo cartilage uptake of the cationic peptidomimetics. Fluorescence images of seven nude mice (post-skin-removal) acquired 2 h post-intravenous injection of 1 nmol of each peptidomimetic probe. Except for Cy5-R_8 and Cy5-$(GO)_7$, strong probe uptake was observed in cartilage-rich locations, including the joints, spine, ears, and ribs.

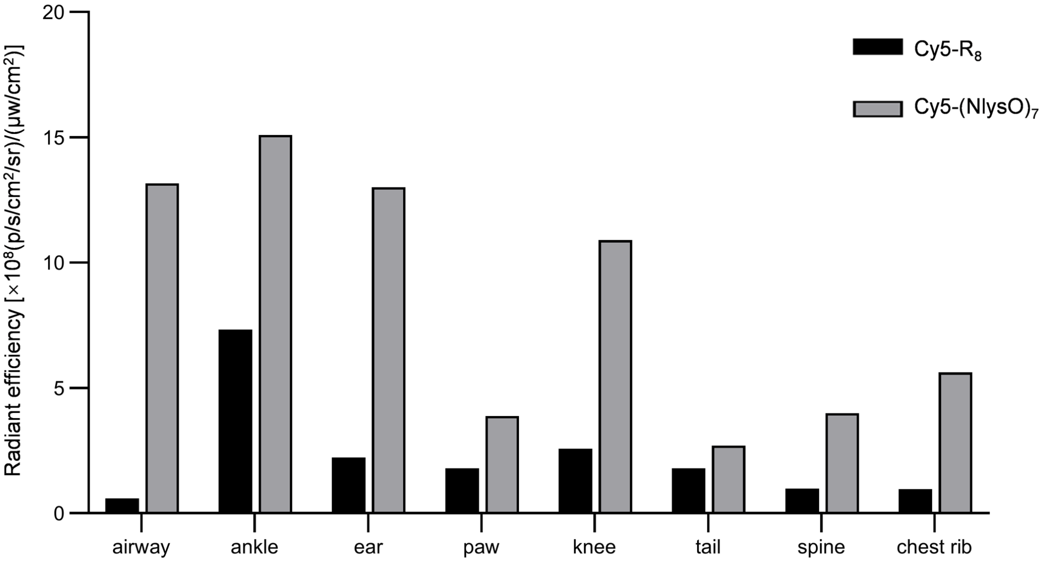

Figure S15. Quantitative analysis of fluorescence signals from the in vivo dosed Cy5-R_8 or Cy5-$(NlysO)_7$ left in the cartilage-rich tissues in Figure 3d.

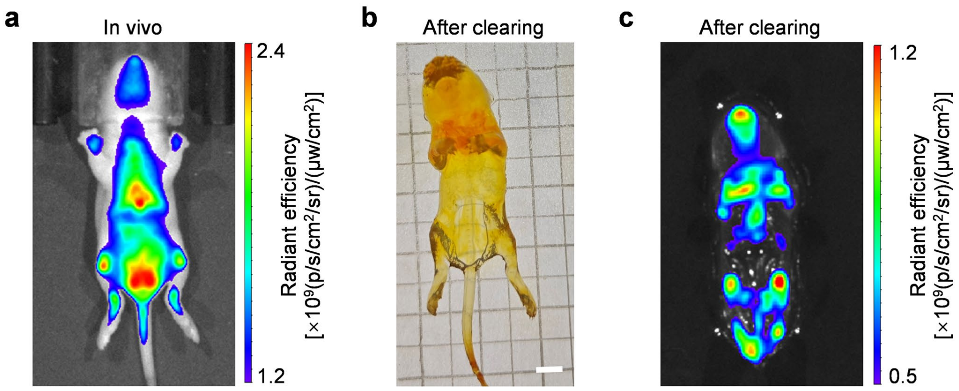

Figure S16. a. A representative in vivo fluorescence image of the neonatal mice (1-week-old, $n = 2$) after tail vein injection of 1 nmol Cy5-(NlysO)$_7$. The signals were largely concentrated in the ribs and joints throughout the body. **b.** A representative bright field photograph of the neonatal mice [pre-injected with Cy5-(NlysO)$_7$] after organ removal and whole-body tissue clearing using the PEGASOS method. **c.** A representative fluorescence image of the cleared mouse body showing strong probe signal retention in the cartilage. Scale bar: 0.5 cm (b).

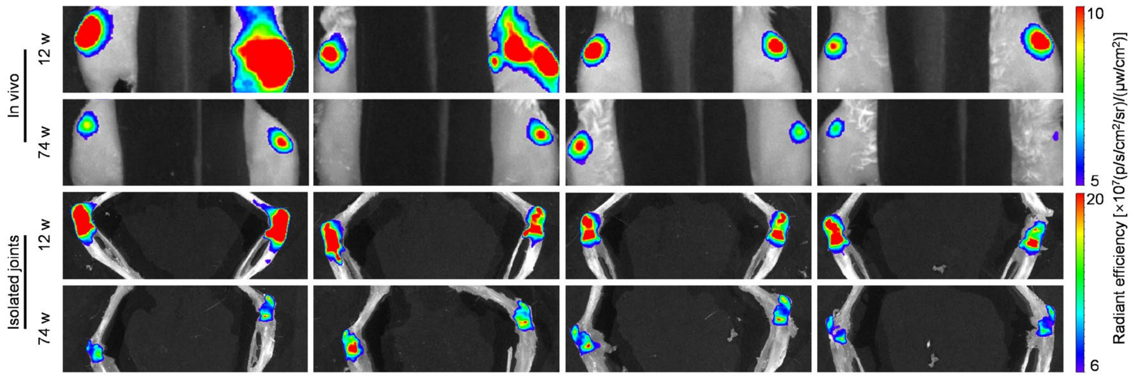

Figure S17. All in vivo and ex vivo fluorescence images showing the Cy5-(NlysO)$_7$ uptake in the knee joints of young (12-week-old) and aged mice (74-week-old) (n = 4 mice). The mice were imaged and sacrificed 2 h post-intravenous injection of 2 nmol Cy5-(NlysO)$_7$. A notable decrease in Cy5-(NlysO)$_7$ uptake in the knee joints of the aged mice suggested possible GAG loss in the articular cartilage.

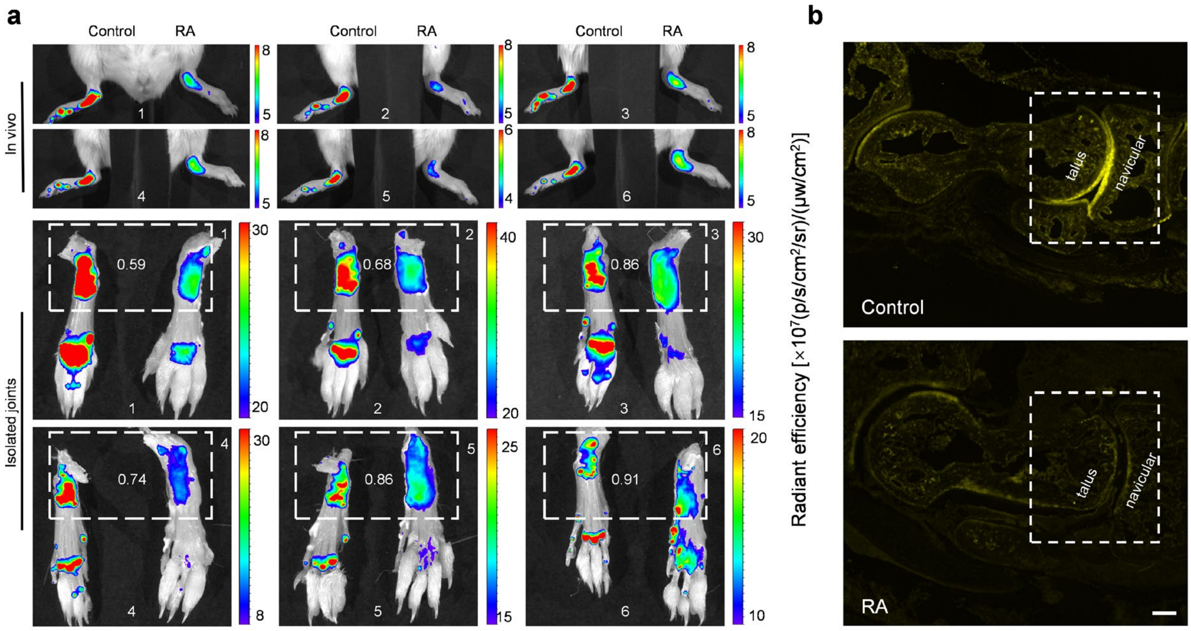

Figure S18. In vivo uptake of Cy5-(NlysO)$_7$ in the inflamed RA ankle joints of the CAIA mice with local single-paw LPS triggering. **a.** All in vivo and ex vivo fluorescence images of the hind paws of the single-paw CAIA model mice (*n* = 6 mice, numbered from 1 to 6) acquired 2 h post intravenous injection of 2 nmol of Cy5-(NlysO)$_7$. The ratio of the quantified Cy5-(NlysO)$_7$ fluorescence signals from the ankle areas between the RA affected and the control hind paws of each mouse was shown inside each dashed box. The results indicated that the fluorescence Cy5-(NlysO)$_7$ uptake in each arthritic paw was significantly decreased compared to its non-arthritic counterpart, presumably due to GAG loss. **b.** Representative fluorescence micrographs of the cryosections of the mice's RA and non-arthritic (control) ankle joints stained with Cy5-(NlysO)$_7$. Scale bar: 100 μm.

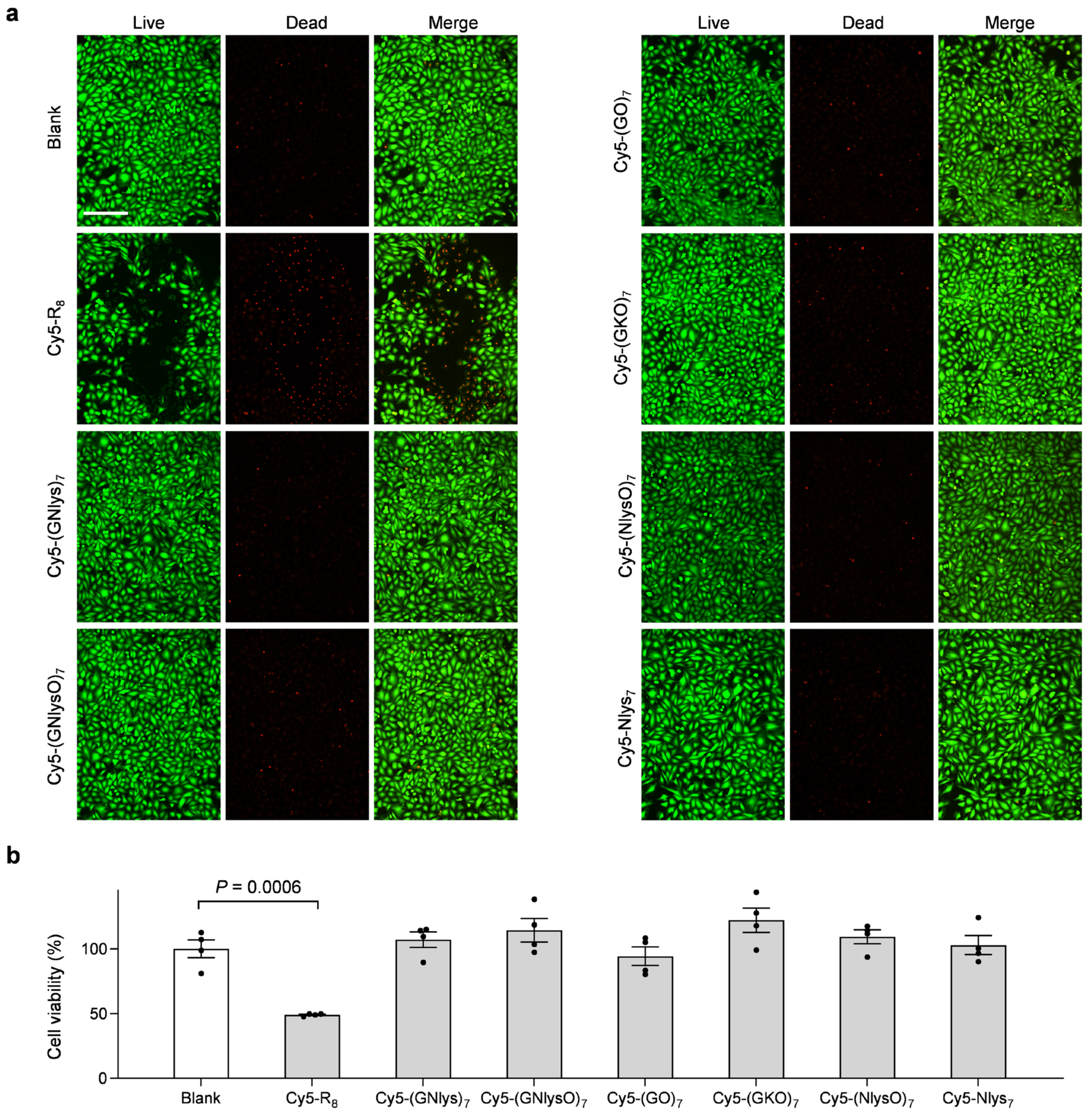

Figure S19. Cytotoxicity of the peptidomimetics assessed by live-dead cell staining. **a**. Fluorescence images of the rat chondrocytes following 24 h of culture in the medium containing 40 μM of each peptidomimetic compound. Green: live cells stained by Calcein-AM. Red: dead cells stained by propidium iodide (PI). **b**. Relative cell viability was estimated from the total fluorescence signals of the images of Calcein-AM-stained live cells of each group (one micrograph per well). Data are displayed as mean ± s.e.m. (data points: $n = 4$ images per group) and analyzed using one-way ANOVA with *post hoc* Tukey HSD test. Scale bar: 275 μm.

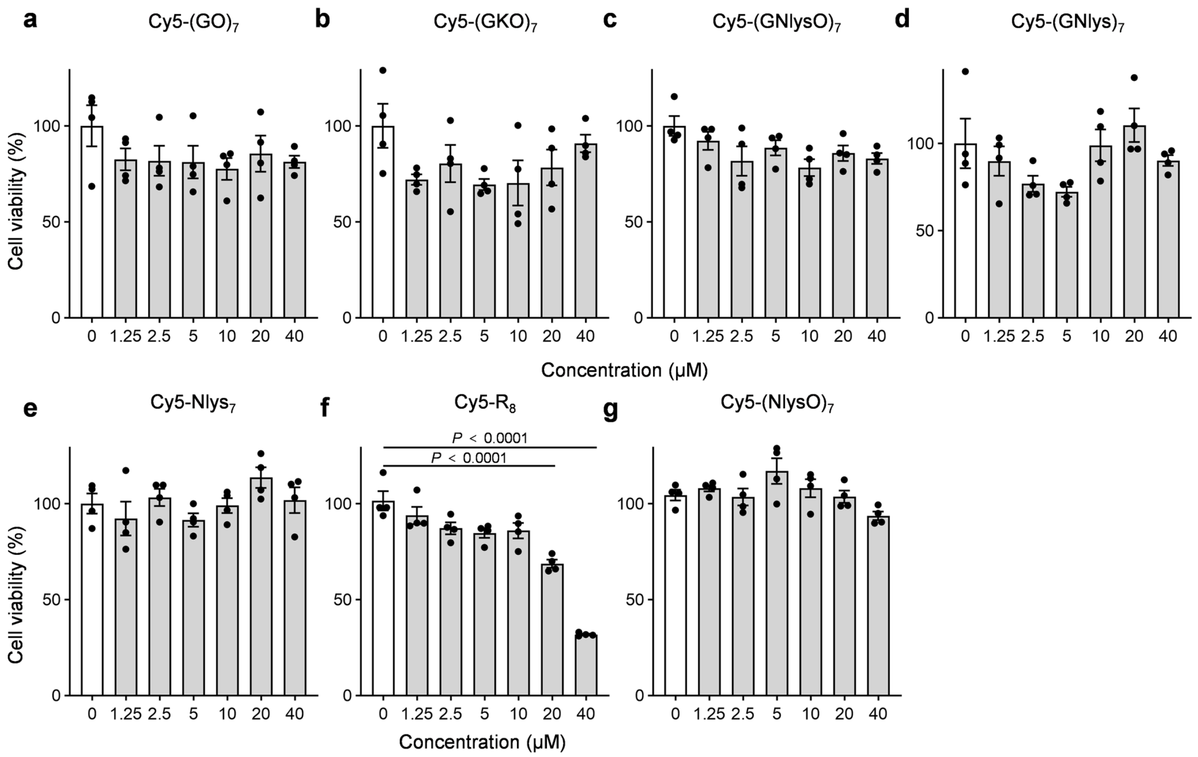

Figure S20. Cytotoxicity of the peptidomimetics assessed by CCK-8 cell viability assays on cultured rat chondrocytes over 24 h. Data are displayed as mean ± s.e.m. (data points: n = 4 individual wells) and analyzed using one-way ANOVA with *post hoc* Tukey HSD test. Except for the Cy5-R_8 group, which exhibited significant cytotoxicity at high concentrations (20-40 μM) compared to the 0 μM control ($P < 0.05$), no compounds showed apparent cytotoxicity at the tested concentrations.

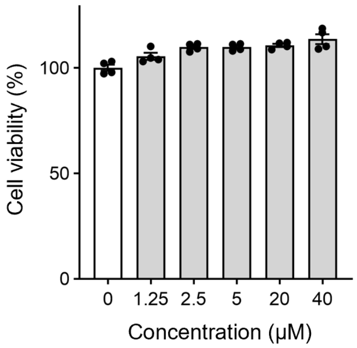

Figure S21. Cytotoxicity of Cy5-$(NlysO)_7$ assessed by CCK-8 cell viability assays on cultured rat chondrocytes over 72 h. Data are displayed as mean ± s.e.m. (data points: $n = 4$ individual wells) and analyzed using the Kruskal-Wallis test with Dunn's multiple comparisons test. None of the Cy5-$(NlysO)_7$ concentration groups differed significantly from the blank (0 μM) control group ($P > 0.05$).

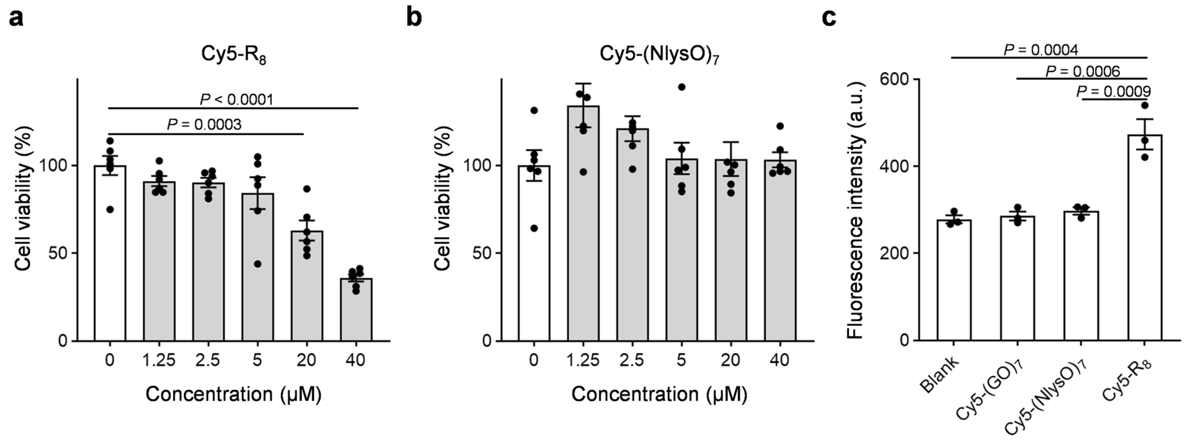

Figure S22. a,b. Cytotoxicity of Cy5-R_8 (a) and Cy5-$(NlysO)_7$ (b) assessed by CCK-8 cell viability assays on cultured human umbilical vein endothelial cells (HUVECs) over 24 h. Data are displayed as mean ± s.e.m. (data points: $n = 6$ individual wells). Numbers were analyzed using one-way ANOVA with *post hoc* Tukey HSD test. Cy5-R_8 exhibited significant cytotoxicity at high concentrations (20-40 µM) compared to the blank (0 µM) control, while Cy5-$(NlysO)_7$ showed no apparent cytotoxicity at the tested concentrations. **c**. Fluorescence intensity of a 96-well plate in which HUVECs were cultured. A plate reader measured the fluorescence intensities after the cells were rinsed following 2 h incubation with no probe (blank), or with 10 µM Cy5-$(GO)_7$, Cy5-$(NlysO)_7$, or Cy5-R_8 in the culture media. Data are displayed as mean ± s.e.m. (data points: $n = 3$ individual wells) and were analyzed using one-way ANOVA with *post hoc* Tukey HSD test. These data demonstrated that the cellular uptake of Cy5-R_8 by the HUVECs was significant, whereas the cellular uptake of Cy5-$(NlysO)_7$ was remarkably low.

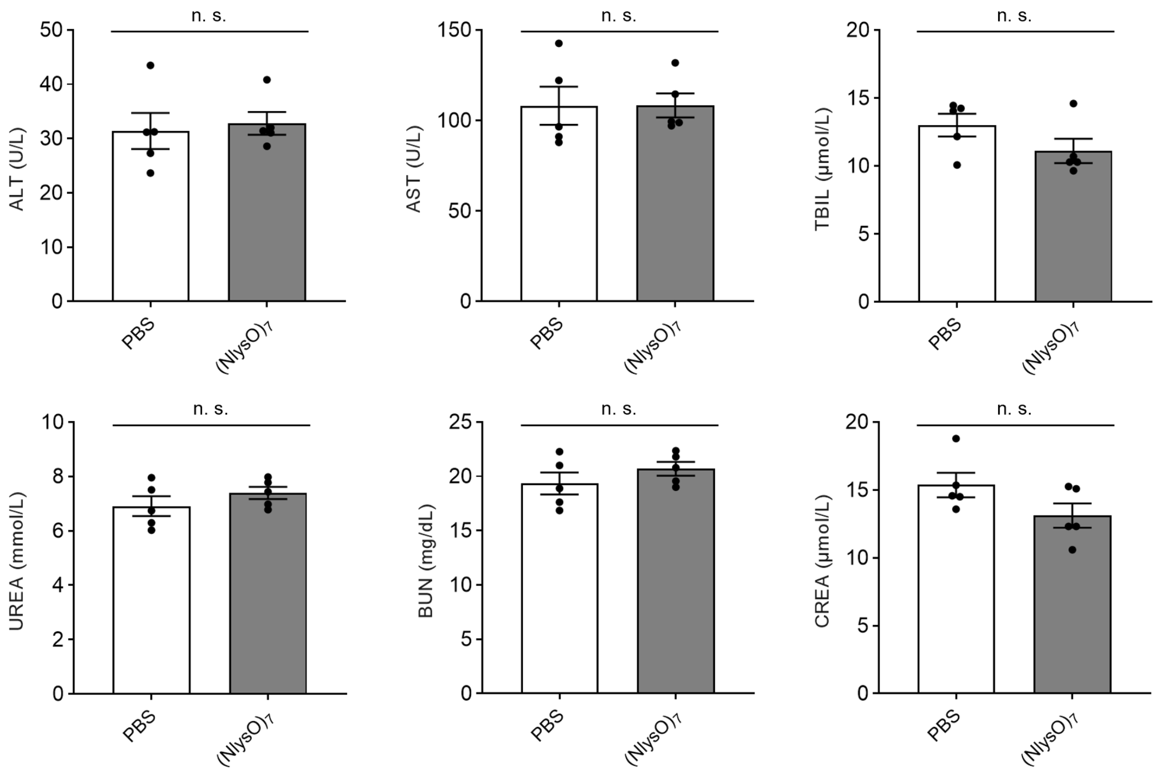

Figure S23. The biochemical analysis of the blood collected from C57BL/6J mice (6–8 weeks old), 24 h post intravenous injection of a blank PBS buffer or 2 nmol of Cy5-(NlysO)$_7$ (n = 5 mice). None of the analytes from the Cy5-(NlysO)$_7$ group significantly differed from those of the PBS buffer control group. Analytes: alanine aminotransferase (ALT), aspartate aminotransferase (AST), total bilirubin (TBIL), urea (UREA), blood urea nitrogen (BUN), creatinine (CREA). Numbers are expressed as mean ± s.e.m. and analyzed using an unpaired t-test. n. s. = not significant ($P > 0.05$).

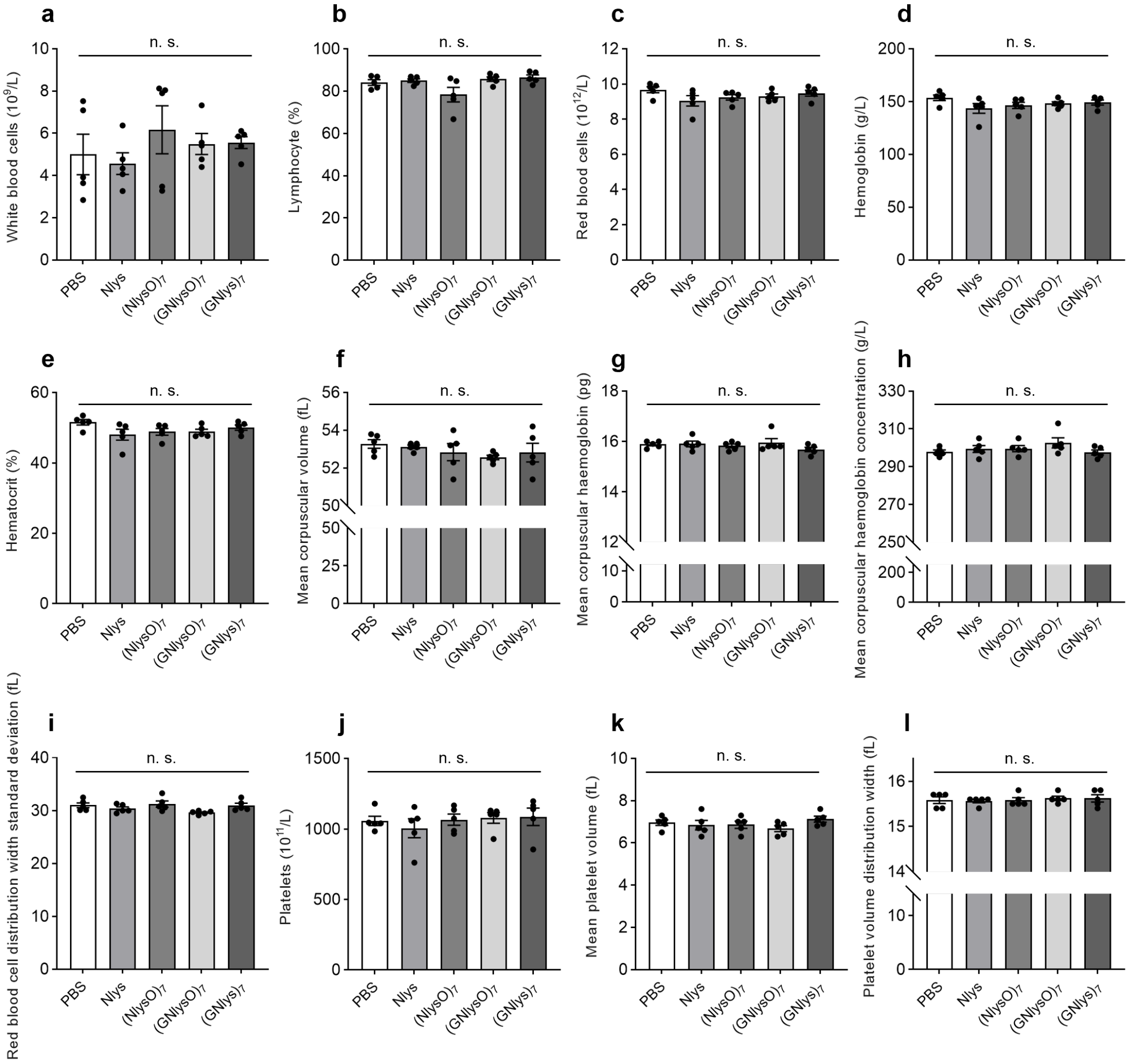

Figure S24. Routine blood analyses for the mice in the PBS, Cy5-Nlys$_7$, Cy5-(NlysO)$_7$, Cy5-(GNlysO)$_7$, and Cy5-(GNlys)$_7$ groups with 3 weekly intravenous doses of PBS buffer or 2 nmol of the tested peptidomimetics over 14 days (n = 5 mice). None of the measurements from the experiment groups significantly differed from those of the PBS control group. Numbers are expressed as mean ± s.e.m. Depending on the distribution and homoscedasticity of the data, the numbers were analyzed using the one-way ANOVA with *post hoc* Tukey HSD test (c-e, h-i and k), Kruskal-Wallis test with Dunn's multiple comparisons test (a, g, j and l) and Welch ANOVA with Dunn's multiple comparisons test (b, f and l). (n. s.: $P > 0.05$).

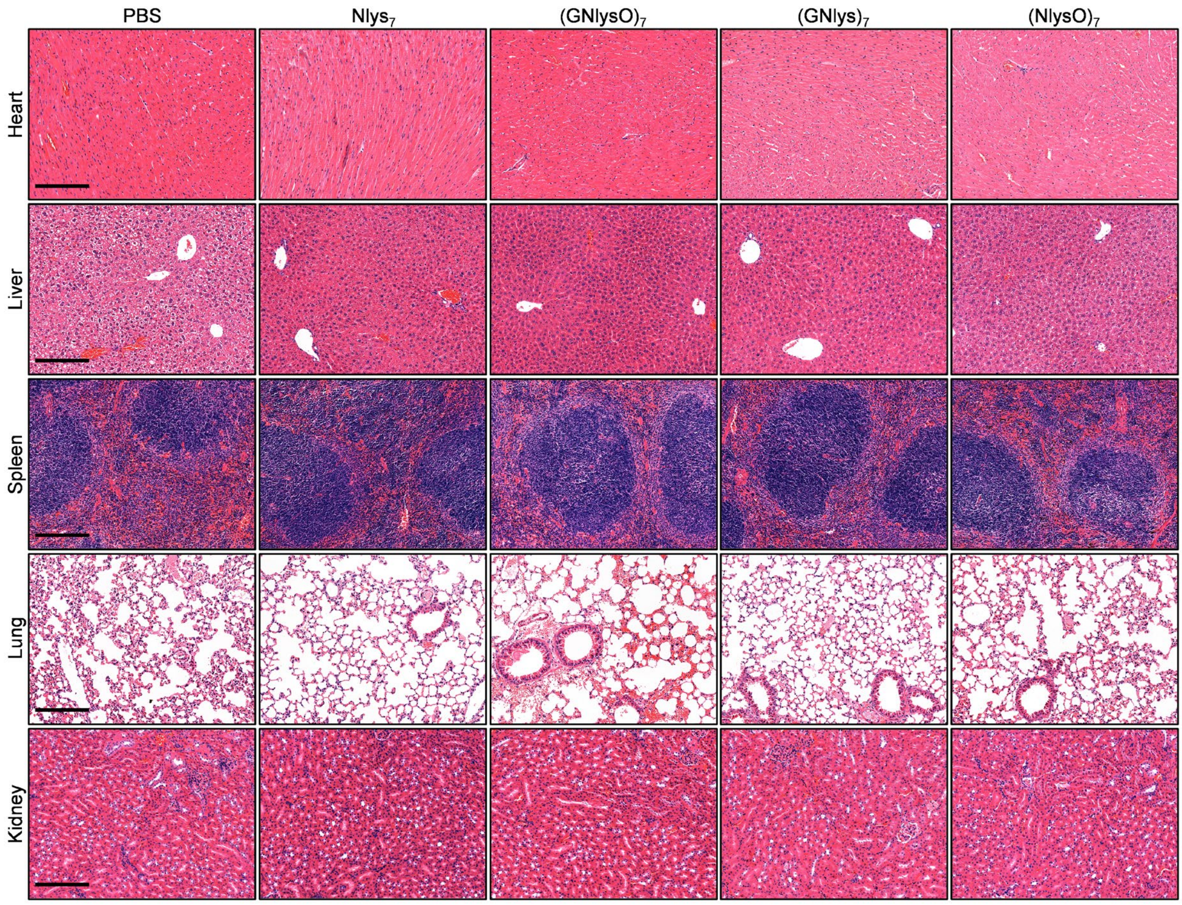

Figure S25. Representative H&E staining of the mice's hearts, livers, spleens, lungs, and kidneys in different groups with 3 weekly intravenous doses of PBS buffer or 2 nmol of the tested peptidomimetics over 14 days (n = 5 mice). Scale bars: 200 μm.

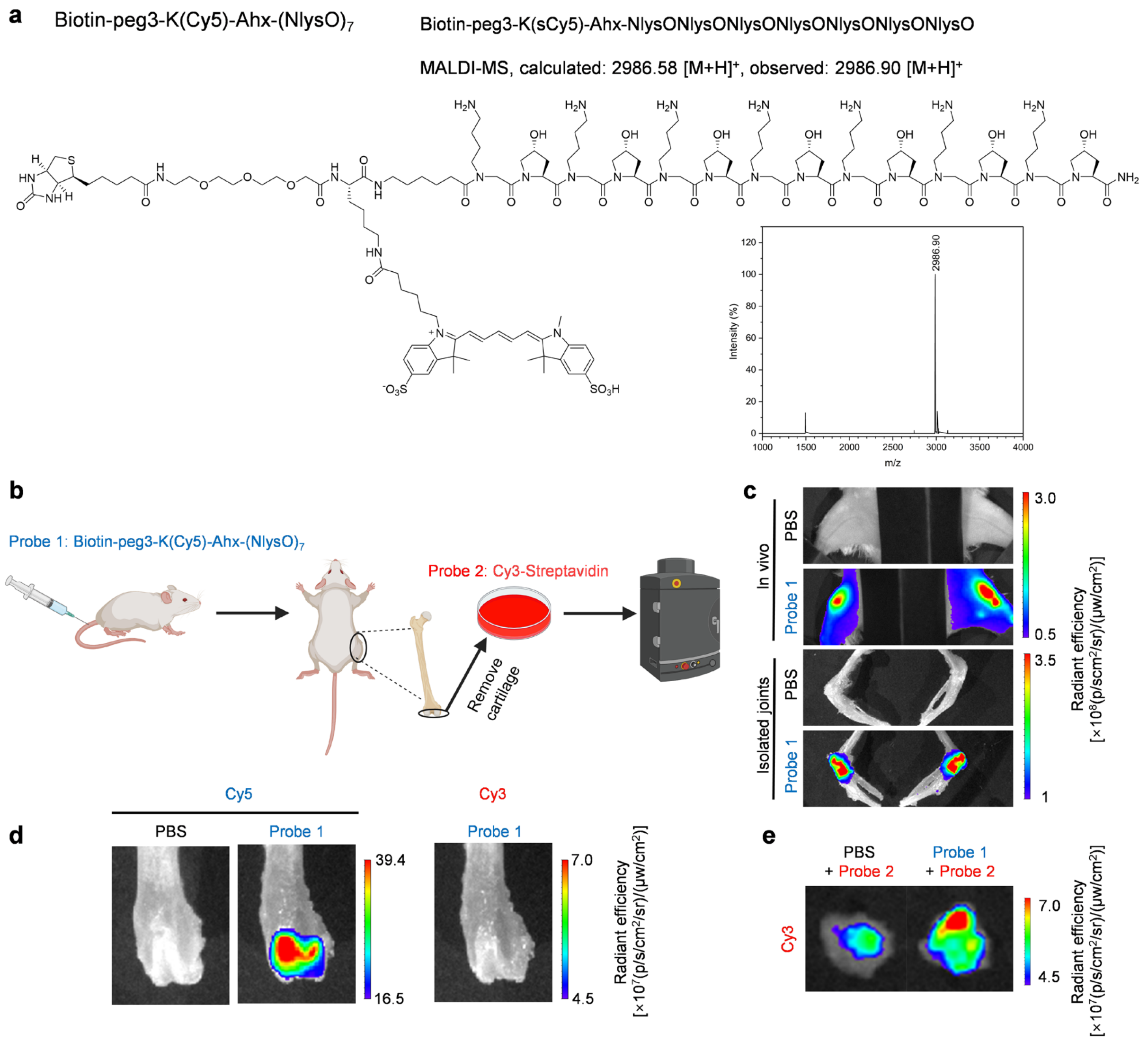

Figure S26. In vivo delivery of biotin as a model drug to the cartilage using $(NlysO)_7$. **a.** The chemical structure and MALDI-MS spectrum of the biotin-$(NlysO)_7$ conjugate biotin-peg3-K(Cy5)-Ahx-$(NlysO)_7$. **b.** Schematic of the in vivo experimental procedure. **c.** The fluorescence images (Cy5 channel) of the mice's knee joints acquired 2 h post-intravenous injection of PBS buffer or 2 nmol biotin-peg3-K(Cy5)-Ahx-$(NlysO)_7$. **d.** The fluorescence images (Cy5/Cy3 channel) of the femurs collected from the mice in b before Cy3-streptavidin staining. **e.** The fluorescence image of the isolated femur cartilage after 2 h of Cy3-streptavidin staining. The stronger Cy3 fluorescence signals from the Cy3-streptavidin bound to the cartilage from the mouse injected with biotin-peg3-K(Cy5)-Ahx-$(NlysO)_7$ indicated the delivery of biotin as a representative drug to the cartilage in vivo.

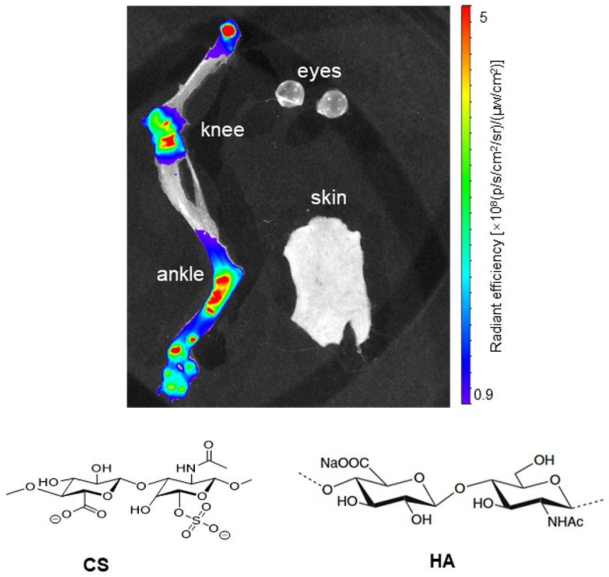

Figure S27. Fluorescence images of the tissues isolated from a mouse 2 h post intravenous injection of 2 nmol Cy5-(NlysO)$_7$. Compared to the strong uptake in the joints, the fluorescence signals from the eyes and skin were almost undetectable, despite their high hyaluronic acid (HA, another GAG type) content. We speculate that this difference could be explained by two aspects. (1) The primary GAG component in the cartilage is chondroitin sulfate (CS). CS contains a disaccharide monomer carrying two negative charges (one from a carboxyl group and the other from a sulfate group). Meanwhile, HA contains only one negative charge per disaccharide unit (from a carboxyl group). Consequently, CS carries twice the number of negative charges compared to HA, enabling it to generate more substantial electrostatic effects. (2) The GAG contents and density of the dense cartilage matrix may be far greater than those of other tissues (e.g., the skin and eyes).

Supplementary Videos

Supplementary Video 1: Neonatal mouse imaging

Light sheet fluorescence microscopy scanning showing the Cy5-$(NlysO)_7$ in vivo binding to the cartilage within the whole body of a 1-week-old neonatal mouse. A dose of 1 nmol Cy5-$(NlysO)_7$ was intravenously injected into the mouse 2 h before specimen collection. Cy5: yellow; autofluorescence: gray. Scale bar: 5 mm.

Supplementary Video 2: Zebrafish imaging

Confocal microscopy scanning shows the in vivo binding of Cy5-$(NlysO)_7$ to the skull cartilage (red) in a zebrafish 5 days post-fertilization. A dose of 0.1 pmol Cy5-$(NlysO)_7$ was intravenously injected into the zebrafish 2 h before imaging. Scale bar: 200 μm.
